# DNA repair-related genes and adipogenesis: Lessons from congenital lipodystrophies

**DOI:** 10.1590/1678-4685-GMB-2022-0086

**Published:** 2022-11-07

**Authors:** Julliane Tamara Araújo de Melo Campos, Matheus Sena de Oliveira, Luisa Pessoa Soares, Katarina Azevedo de Medeiros, Leonardo René dos Santos Campos, Josivan Gomes Lima

**Affiliations:** 1 Universidade Federal do Rio Grande do Norte, Departamento de Biologia Celular e Genética, Laboratório de Biologia Molecular e Genômica, Natal, RN, Brazil.; 2 Universidade Federal do Rio Grande do Norte, Faculdade de Ciências da Saúde do Trairi, Santa Cruz, RN, Brazil.; 3 Universidade Federal do Rio Grande do Norte, Bioinformatics Multidisciplinary Environment, Natal, RN, Brazil.; 4 Universidade Federal do Rio Grande do Norte, Departamento de Medicina Clínica, Hospital Universitário Onofre Lopes, Natal, RN, Brazil.

**Keywords:** DNA repair, adipogenesis, genetic lipodystrophies, metabolism

## Abstract

Classical and progeroid congenital lipodystrophies are a collection of rare diseases displaying a large genetic heterogeneity. They occur due to pathogenic variants in genes associated with adipogenesis, DNA repair pathways, and genome stability. Subjects with lipodystrophy exhibit an impairment in the homeostasis of subcutaneous white adipose tissue (sWAT), resulting in low leptin and adiponectin levels, insulin resistance (IR), diabetes, dyslipidemia, ectopic fat deposition, inflammation, mitochondrial and endoplasmic reticulum commitments, among others. However, how pathogenic variants in adipogenesis-related genes modulate DNA repair in some classical congenital lipodystrophies has not been elucidated. In the same way, no data is clarifying how pathogenic variants in DNA repair genes result in sWAT loss in different types of progeroid lipodystrophies. This review will concentrate on the main molecular findings to understand the link between DNA damage/repair and adipogenesis in human and animal models of congenital lipodystrophies. We will focus on classical and progeroid congenital lipodystrophies directly or indirectly related to DNA repair pathways, highlighting the role of DNA repair-related proteins in maintaining sWAT homeostasis.

## Introduction

Nuclear and mitochondrial DNA are continuously exposed to damage induced by endogenous and exogenous sources (Evans *et al.*, 2004; Bauer *et al.*, 2015). Endogenous sources of DNA damage include reactive oxygen species (ROS) generated during normal cell metabolism, mainly by the mitochondria (Balaban *et al.*, 2005), but also by the endoplasmic reticulum (ER), peroxisomes, and cell membrane (Bhattacharyya *et al.*, 2014). Furthermore, exogenous DNA damage sources mainly include ultraviolet (UV) radiation, ionizing radiation (IRa),** **and alkylating agents (Evans **et al.*,* 2004). 

Cells have developed several DNA repair pathways to defend the genome against different types of damage, including the most deleterious lesions, such as oxidized DNA lesions, single strand breaks (SSBs), and double-strand breaks (DSBs) (Limpose *et al.*, 2017). DNA repair pathways protect from frequent lesions resulting in DNA breaks. Oxidized DNA lesions and SSBs are usually repaired by the base excision repair (BER); DSBs are repaired by homologous recombination (HR) and non-homologous end joining (NHEJ). Although nucleotide excision repair (NER) is mainly responsible for repairing bulky DNA-distorting lesions induced by UV radiation, this pathway is also involved with the repair of oxidized DNA lesions together with BER (Dianov *et al.*, 1999; Stevnsner *et al.*, 2002; Tuo *et al.*, 2002; D’Errico *et al.*, 2006; Stevnsner **et al.*,* 2008; de Melo *et al.*, 2016; Kumar *et al.*, 2020). There are two NER sub-pathways, global genomic-NER (GG-NER) and transcription-coupled NER (TC-NER), which differ only in the initial step of DNA lesion recognition. 

Failure to repair DNA damage or misrepaired DNA lesions leads to genomic instability and changes in cellular homeostasis, resulting in cancer (Menck and Munford, 2014; Jeggo *et al.*, 2016), neurodegenerative diseases (Weissman *et al.*, 2007; Krasikova *et al.*, 2021), aging (Schumacher *et al.*, 2021), and progeroid diseases with loss of subcutaneous white adipose tissue (sWAT) (López-Otín *et al.*, 2013; Araújo-Vilar and Santini, 2019; Araújo de Melo Campos *et al.*, 2021). For example, in the progeroid Cockayne Syndrome (CS), defects in NER may lead to premature aging with loss of sWAT (László and Simon, 1986; Nance and Berry, 1992; Kamenisch *et al.*, 2010). Aging is a process that disturbs most living cells and is related to the accretion of damage to the molecules, genomic instability, telomere dysfunction, heterochromatin loss, and loss of sWAT. Other hallmarks of aging include mitochondrial dysfunction, senescence, inflammation, deregulated nutrient sensing, and metabolic defects. Altogether, these changes lead to a failure in stem cell function, reducing their capabilities to regenerate tissue (Schosserer *et al.*, 2018; Palmer *et al.*, 2019; Smith *et al.*, 2021). 

Over the past decade, a renewed interest in adipose tissue functions and genomic integrity has emerged. Accumulation of senescent white adipocytes occurs during aging, which is associated with hypertrophy of adipocytes, dyslipidemia, and IR (Unger, 2005; Smith *et al.*, 2021; Von Bank *et al.*, 2021). Extreme decrease of sWAT and senescence of adipocytes are hallmarks of an advanced age (Tchkonia *et al.*, 2010; Liu *et al.*, 2018). During aging, the reduced capacity of sWAT to store lipids may contribute to metabolic complications due to ectopic deposition of lipids (lipotoxicity) (Von Bank, **et al.*,* 2021). The mechanisms involved in adipose tissue aging were recently reviewed (Ou *et al.*, 2022). The main hallmarks of senescent cells are a secretory phenotype, cell cycle arrest, and activation of a DNA damage response (DDR), with phosphorylated histone H2AX (γ-H2AX) and p53 expression as markers of senescent cells (Tchkonia **et al.*,* 2010; Liu **et al.*,* 2018). Further, a lower expression of the *H2AX* gene was found in sWAT of obese individuals (Rohde **et al.*,* 2020). However, the link between senescence, DNA damage, and loss of sWAT in congenital lipodystrophies is poorly understood.

This review discusses recent molecular findings in the study of congenital lipodystrophies and the role of DNA repair in maintaining adipose tissue’s functions. We focused on human and animal models of congenital lipodystrophies to unravel the link between DNA damage/repair and sWAT homeostasis.

### sWAT physiology and aging

White adipose tissue **(**WAT) has been extensively studied due to the association between increased visceral WAT (vWAT) and metabolic and cardiovascular disturbs (Tchkonia *et al.*, 2010; Item and Konrad, 2012). On the contrary, studies concerning sWAT and brown adipose tissue (BAT) have shown their beneficial effects in improving metabolism and insulin sensitivity. These findings highlight that distinct WAT depots have different roles related to metabolic health. While vWAT is found around visceral organs, such as gonadal, retroperitoneal, perirenal, omental, and mesenteric localization, depots of sWAT have restricted localization and functions, being found mainly under the skin (metabolically active sWAT) and in palms and soles (mechanic sWAT) (Wajchenberg, 2000; Choe *et al.*, 2016; Schosserer *et al.*, 2018). 

The primary interest of studies concerning WAT physiology was mainly directed to its role as an energy storage tissue. However, over the last years, WAT research has gained a lot of attention since WAT has an essential hormonal function and undergoes significant changes during aging (Ou *et al.*, 2022). One of the proposed aging hallmarks is dysfunctional adipose tissue and the consequent metabolic defects, including a reduction in the levels of somatotrophic axis hormones, such as insulin-like growth factor 1 (IGF1) and growth hormone (GH), as well as steroid hormones (Carrero *et al.*, 2016). Indeed, changes in redox homeostasis have been found in metabolic syndrome, obesity, type 2 diabetes mellitus (DM), and lipodystrophies. During aging, WAT suffers redistribution, BAT depots decrease, and adipose progenitor and stem cells (APSCs) decline. Further, dysfunctional smaller cells similar to adipocytes increase in aged WAT, which show reduced insulin sensitivity than fully differentiated adipocytes (Kirkland *et al.*, 2002). Altogether, these age-related changes in adipose tissue result in decreased sWAT and increased vWAT depots, compromising body function. The pathophysiology of adipose tissue in lipodystrophies was remarkably discussed in recent reviews (Zammouri *et al.*, 2021; Lim *et al.*, 2021; Le Lay *et al.*, 2022).

### Classical and progeroid congenital lipodystrophies

Genetic lipodystrophies are a group of rare, heterogeneous metabolic diseases caused by a lack of sWAT, which can be total or partial (Garg, 2011; Brown *et al.*, 2016; Zammouri *et al.*, 2021; Araújo de Melo Campos *et al.*, 2021). As in aging, congenital lipodystrophies have been associated with adipose tissue redistribution, sWAT loss, increased vWAT, and ectopic fat deposition (Garg and Agarwal, 2009; Zammouri **et al.*,* 2021). The nearly complete lack of body fat at birth results in Congenital Generalized Lipodystrophy (CGL), the most severe form of lipodystrophy. Instead, Familial Partial Lipodystrophy (FPLD) is characterized by a deficiency of sWAT in the limbs and gluteus that emerges during childhood or puberty, associated with fatty tissue deposition in specific body regions, such as the face, neck, and intra-abdominal area. Progeroid syndromes are also a group of rare congenital diseases characterized by clinical features including aging, hair loss, cardiovascular commitments, comorbidities affecting the skeleton and muscle, lipodystrophy, metabolic changes, and others (Van Der Pluijm *et al.*, 2007; Turaga *et al.*, 2009; Carrero *et al.*, 2016). Since generalized or partial lipodystrophy is an important clinical finding associated with numerous progeroid diseases, treatment strategies have been developed to fight metabolic and mitochondrial commitments found in these syndromes (Carrero **et al.*,* 2016; Zammouri **et al.*,* 2021). In this review, we will focus only on classical and progeroid lipodystrophies associated with senescence, DNA damage accumulation, and metabolic dysfunction, three hallmarks of aging (López-Otín *et al.*, 2013). Table 1 shows the main classical and progeroid congenital syndromes.

**Table 1 -:** The mains progeroid and classical inherited lipodystrophies.

Gene code (*OMIM code)	Protein name	Disease name / Degree of sWAT loss	Disease code (#OMIM code)	Adipose tissue commitment	Inheritance	Reference
Progeroid inherited lipodystrophies due to pathogenic variants mainly in genes associated with DNA repair and genomic stability.						
*POLR3A* (*614258)	Polymerase III, RNA, subunit A	Wiedemann-Rautenstrauch Syndrome / Partial	WDRTS (#264090)	Progressive generalized loss of sWAT.	AR	(Rautenstrauch *et al.*, 1977; Wiedemann, 1979)
*FBN1* (*134797)	Fibrillin-1	Marfan Lipodystrophy Syndrome / Partial	MFLS (#616914)	Severe loss of sWAT.	AD	(Horn & Robinson, 2011; Takenouchi *et al.*, 2013)
*RECQL2* (*604611)	DNA helicase, RECQ protein-like 2	Werner Syndrome / Partial	WRN (#277700)	Loss of sWAT.	AR	(Werner, 1985; Huang *et al.* , 2006)
*RECQL3* (*604610)	DNA helicase, RECQ protein-like 3	Bloom Syndrome / Partial	BLM (#210900)	Loss of sWAT.	AR	(Bloom, 1954; Diaz *et al.*, 2006)
*LMNA* (*150330)	Lamin-A/C	Type A Mandibuloacral Dysplasia with Lipodystrophy / Partial	MADA (#248370)	Partial loss of sWAT.	AR	(Young *et al.*, 1971)
*ZMPSTE24* (*606480)	Zinc metalloproteinase STE24 homolog	Type B Mandibuloacral Dysplasia with Lipodystrophy / Generalized	MADB (#608612)	Generalized loss of sWAT.	AR	(Agarwal *et al.*, 2003a)
*BANF1* (*603811)	Barrier-to-autointegration factor	Néstor-Guillermo Progeria Syndrome / Generalized	NGPS (#614008)	Generalized loss of sWAT.	AR	(Puente *et al.*, 2011)
*SPRTN* (*616082)	DNA-Dependent Metalloprotease Spartan	Ruijs-Aalfs Syndrome / Generalized	RJALS (#616200)	Loss of sWAT.	AR	(Ruijs *et al.*, 2003; Lessel *et al.*, 2014)
*ERCC8* (*609412)	Excision repair cross-complementing, group 8	Type A Cockayne Syndrome / Generalized	CSA (#216400)	Progressive loss of sWAT.	AR	(Nance & Berry, 1992; Brace *et al.*, 2013; Brace **et al.*,* 2016)
*ERCC6* (*609413)	Excision repair cross-complementing, group 6	Type B Cockayne Syndrome / Generalized	CSB (#133540)	Generalized sWAT loss.	AR	(Nance & Berry, 1992; Van Der Pluijm *et al.*, 2007)
*ERCC4* (*133520)	Excision repair cross-complementing, group 4	Xeroderma Pigmentosum Complementation Group F	XPF (#278760)	Generalized sWAT loss.	AR	(Niedernhofer *et al.*, 2006)
*LMNA* (*150330)	Lamin-A/C	Hutchinson-Gilford Progeria Syndrome / Generalized	HGPS (#176670)	sWAT loss.	AD	(Hutchinson, 1886; Goldman *et al.*, 2004)
*CAV1* (*601047)	Caveolin-1	Severe premature aging and lipodystrophy	-	Generalized sWAT loss.	AR	(Schrauwen *et al.*, 2015; Garg *et al.*, 2015)
*POLD1* (*174761)	DNA polymerase delta 1	Mandibular hypoplasia, deafness, progeroid features, and lipodystrophy syndrome.	MDPL (#615381)	Prominent loss of sWAT.	AD	(Shastry *et al.*, 2010; Weedon *et al.*, 2013)
Classical inherited lipodystrophies due to pathogenic variants mainly in genes associated with adipogenesis.						
*LMNA* (*150330)	Lamin-A/C	Type 2 Familial Partial Lipodystrophy / Partial	FPLD2 (#151660)	Partial loss of sWAT.	AD	(Caron *et al.*, 2007)
*PPARG* (*601487)	PPARγ	Type 3 Familial Partial Lipodystrophy / Partial	FPLD3 (#604367)	Partial loss of sWAT.	AD	(Agarwal and Garg, 2002; Hegele *et al.*, 2002)
*PLIN1* (*170290)	Perilipin-1	Type 4 Familial Partial Lipodystrophy / Partial	FPLD4 (#613877)	Partial loss of sWAT.	AD	(Gandotra *et al.*, 2011)
*CIDEC* (*612120)	Cell Death Inducing DFFA like Effector C	Type 5 Familial Partial Lipodystrophy / Partial	FPLD5 (#615238)	Partial loss of sWAT.	AR	(Rubio-Cabezas *et al.*, 2009)
Classical inherited lipodystrophies due to pathogenic variants mainly in genes associated with adipogenesis.						
*LIPE* (*151750)	Hormone-sensitive lipase (HSL)	Type 6 Familial Partial Lipodystrophy / Partial	FPLD6 (#615980)	Partial loss of sWAT.	AR	(Albert *et al.*, 2014; Zolotov *et al.*, 2017)
*CAV1* (*601047)	Caveolin-1	Type 7 Familial Partial Lipodystrophy / Partial	FPLD7 (#606721)	Partial loss of sWAT.	AD	(Cao *et al.*, 2008)
*MFN2* (*608507)	Mitofusin 2	Multiple Symmetric Lipomatosis / Partial	MSL (#151800)	Partial loss of sWAT.	AR	(Capel *et al.*, 2018)
*AGPAT2* (*603100)	1-AGPAT 2	Type 1 Berardinelli-Seip Congenital Lipodystrophy / Generalized	CGL1 (#608594)	Generalized loss of sWAT.	AR	(Agarwal *et al.*, 2002)
*BSCL2* (*606158)	Seipin	Type 2 Berardinelli-Seip Congenital Lipodystrophy / Generalized	CGL2 (#269700	Generalized loss of sWAT.	AR	(Magré *et al.*, 2001)
*CAV1* (*601047)	Caveolin-1	Type 3 Berardinelli-Seip Congenital Lipodystrophy / Generalized	CGL3 (#612526)	Generalized loss of sWAT.	AR	(Kim *et al.*, 2008)
*CAVIN1* (*603198)	Cavin-1	Type 4 Berardinelli-Seip Congenital Lipodystrophy / Generalized	CGL4 (#613327)	Generalized loss of sWAT.	AR	(Hayashi *et al.*, 2009; Rajab *et al.*, 2010)

OMIM: Online Mendelian Inheritance in Man. AR: Autosomal recessive. AD: Autosomal dominant.

### Congenital generalized lipodystrophies - CGLs

The lack of sWAT in CGL causes a decrease in leptin levels and alters food intake, intensifying the appetite (Badman & Flier, 2007; Rodríguez *et al.*, 2016). The blood circulating lipids result in hypertriglyceridemia (HTG), and their accumulation in ectopic sites, such as in the liver and skeletal muscle, can result in hepatic steatosis and weakness of respiratory muscle strength, respectively (Debray *et al.*, 2013; Dantas De Medeiros **et al.*,* 2018; Araújo de Melo Campos *et al.*, 2021). Severe IR causes hypertension, HTG, and difficulty in controlling diabetes. Liver fat deposition can result in cirrhosis. These comorbidities could explain the severity of CGL and its early mortality (Lima *et al.*, 2018b). 

The most common pathogenic variants associated with CGLs are in *AGPAT2* and *BSCL2* genes, related to types 1 and 2 (CGL1 and CGL2), respectively (Magré *et al.*, 2001; Agarwal *et al.*, 2002; Craveiro Sarmento *et al.*, 2019). Although CGL1 and CGL2 have similar metabolic abnormalities, the sWAT loss is less severe in CGL1 individuals, which have more mechanical sWAT, while CGL2 individuals display a significant reduction of both metabolically active and mechanic sWAT (Garg *et al.*, 1992; Agarwal **et al.*,* 2003b; Simha and Garg, 2003). Regarding the *AGPAT2* gene, it codifies to the 1-acylglycerol-3-phosphate o-acyltransferase (1-AGPAT 2) enzyme, which is associated with the synthesis of triacylglycerol (TG) and phospholipids in the ER (Agarwal and Garg, 2003). Recessive pathogenic variants in the *BSCL2* gene, which codifies to the ER membrane-localized seipin, are the genetic cause of CGL2 (Magré **et al.*,* 2001). This protein acts to regulate the TG transport from the ER to lipid droplets (LDs) (Salo *et al.*, 2019), converting nascent to mature LDs (Wang *et al.*, 2016) and regulating ER-LDs contacts and cargo delivery (Salo **et al.*,* 2016). Seipin has essential functions related to adipose tissue homeostasis, such as coordinating 1-AGPAT2 function (Sim **et al.*,* 2020) and controlling Ca^2+^ (calcium) import and adipocyte metabolism at ER-mitochondria sites (Combot *et al.*, 2022). 

Type 3 CGL (CGL3) occurs due to homozygous pathogenic variants in the *CAV1* gene that codifies to caveolin-1 (Kim *et al.*, 2008), whereas type 4 CGL (CGL4) occurs due to pathogenic variants in the *CAVIN1* gene, which codifies to the cavin-1 protein (Hayashi *et al.*, 2009; Rajab *et al.*, 2010). Both cavin-1 and caveolin-1 are present in caveolae, which are cave-like structures located at the plasma membrane in most cells, mainly adipocytes. Caveolae are involved in cellular processes, such as cell metabolism, cholesterol homeostasis, cell proliferation, and senescence (Parton, 2018). However, the number of pathogenic variants in both genes is scarce relative to CGL1 and CGL2. Table 1 contains a summary of the the molecular basis and sWAT physiology of CGL syndromes.

At the morphological level, CGL subjects present a typical phenotype, revealing acromegalic facies, prominent musculature, prognathism, phlebomegaly (prominent veins), umbilical protrusion, *acanthosis nigricans*, acrochordons, hirsutism, bone cysts, and others (Garg, 2000; Maldergem *et al.*, 2002; Agarwal *et al.*, 2003b; Garg and Agarwal, 2009; Vigouroux *et al.*, 2011; Lima *et al.*, 2016; Lima **et al.*,* 2017; Lima **et al.*,* 2018a). At metabolic and physiological levels, CGL subjects present dyslipidemia, hyperinsulinemia, IR, DM, low levels of leptin and adiponectin, decreased levels of high-density lipoprotein cholesterol (HDL-c), hepatosplenomegaly, and hypertrophic cardiomyopathy (Faria *et al.*, 2009; Lima **et al.*,* 2016; de Azevedo Medeiros *et al.*, 2017; Ponte *et al.*, 2018; Dantas De Medeiros **et al.*,* 2018). 

### Familiar partial lipodystrophies - FPLDs

Concerning the FPLDs, eight subtypes were described, and the primary molecular causes of these heterogeneous diseases are genes related to the nuclear envelope and adipocyte homeostasis, such as *LMNA* and *PPARγ* (Patni and Garg, 2015; Araújo-Vilar and Santini, 2019; Fernández-Pombo *et al.*, 2021). Type 1 FPLD (FPLD1, also named Köbberling syndrome) is probably a multigenic form of lipodystrophy (Patni and Garg, 2015; Araújo-Vilar and Santini, 2019). The most frequent FPLD is the Dunnigan syndrome, also referred to as type 2 FPLD (FPLD2), which occurs due to pathogenic variants in the *LMNA* gene. This gene encodes lamin-A and lamin-C (besides lamins CΔ10 and C2) which play a significant function in maintaining the stability of the cellular nucleus by physically supporting nuclear envelope components (Gonzalo *et al.*, 2017). Over 400 pathogenic variants were described in the *LMNA* gene. In addition to FPLD2, they are related to more than a dozen degenerative diseases, such as neuropathies, muscular dystrophies, and premature aging (Broers **et al.*,* 2006; Bertrand *et al.*, 2011; Gonzalo and Kreienkamp, 2015). Recent reviews discussed the association between *LMNA* variants and several diseases (Ho and Hegele, 2019; Lazarte and Hegele 2021). However, how different *LMNA* pathogenic variants result in a plethora of diseases has yet to be unraveled. 

FPLD2 phenotype was initially described in 1974 by Dunnigan and first associated with the *LMNA* gene in 1998 by Peters *et al.* (Dunnigan **et al.*,* 1974; Peters **et al.*,* 1998). This disease is characterized by loss of sWAT in the extremities and trunk, sparing the face and neck at puberty. Lamins A/C, encoded by the *LMNA* gene, are nuclear proteins, and specific pathogenic variants may lead to nuclear function disruption, resulting in premature adipocyte death (Garg, 2011). FPLD2 subjects show loss of sWAT mainly in the axial skeleton, such as in limbs, trunk, hips, and gluteus, but not in the appendicular skeleton (Garg **et al.*,* 2001; Chan *et al.*, 2016). FPLD2 metabolic disturbances include HTG, low HDL-c levels, IR, hepatic steatosis, pancreatitis, and a high probability of developing cardiovascular diseases (Araújo-Vilar and Santini, 2019; Lazarte *et al.*, 2021). 

Type 3 (FPLD3) is caused by pathogenic variants in the *PPAR*γ gene. In 1999, three subjects were reported with severe IR harboring two different heterozygous pathogenic variants in the ligand-binding domain of peroxisome proliferator-activated receptor type γ (PPARγ) (Barroso *et al.*, 1999). Later, these variants were associated with FPLD3 (Savage *et al.*, 2003). As PPARγ is a critical transcription factor for adipogenesis, its dominant pathogenic variants may impair adipocyte differentiation (Garg, 2011). This type is characterized by loss of sWAT in the extremities, especially in distal regions (Araújo-Vilar and Santini 2019).

Type 4 FPLD (FPLD4) was described and associated with two distinct heterozygous frameshift pathogenic variants in the *PLIN1* gene (Gandotra *et al.*, 2011). The PLIN1 gene encodes perilipin-1, an integral component of LDs, playing an essential role in lipid storage and hormone-regulated lipolysis (Garg, 2011). In this type, lipoatrophy is mainly evident in the gluteal regions and lower limbs, although the loss of subcutaneous fat has also been observed in the trunk and upper limbs. 

Type 5 FPLD (FPLD5) is caused by a homozygous truncating pathogenic variant in the *CIDEC* gene that was first reported in 2009 (Rubio-Cabezas *et al.*, 2009). The clinical hallmarks are loss of sWAT in the lower limbs, prominent muscle mass, IR, diabetes, and decreased LD size in adipocytes. The *CIDEC* gene encodes the Cell Death Inducing DFFA Like Effector C (CIDEC) protein that is associated with LDs, inhibiting lipolysis and promoting the formation of unilocular LDs in adipocytes (Garg, 2011).

Type 6 FPLD (FPLD6) is triggered by a homozygous pathogenic variant in the *LIPE* gene. The first to describe this disease and its association with this gene were Albert *et al.* (2014). The main clinical manifestations of this disease are progressive loss of sWAT in the legs that correlate with abnormal fat distribution, including fat accumulation in the neck, face, axilla, shoulders, back, abdomen, and pubic region. Furthermore, in some cases, myopathy, diabetes, HTG, low HDL-c, and hepatic steatosis may be observed (Zolotov *et al.*, 2017).

Pathogenic variants in the *CAV1* gene, first related to CGL3, were also found in type 7 FPLD (FPLD7) individuals (Cao *et al.*, 2008). However, heterozygous pathogenic variants in this gene are responsible for causing FPLD7 (Cao **et al.*,* 2008). This disease is characterized by loss of sWAT in different regions of the body, accompanied by metabolic complications such as IR, lipid abnormalities, and in some cases, cataracts and muscle spasticity (Garg *et al.*, 2015). More studies are required to unravel the role of distinct *CAV1* pathogenic variants in different types of congenital lipodystrophies, such as CGL3, FPLD7, and the neonatal onset of generalized lipodystrophy (Cao **et al.*,* 2008; Schrauwen *et al.*, 2015; Garg **et al.*,* 2015). Table 1 summarizes the molecular basis and sWAT physiology of FPLD syndromes.

### Progeroid disorders

Monogenic, premature aging diseases are heterogeneous syndromes and present variable severity and overlapping phenotypes, making it difficult for the correct clinical diagnosis (Carrero *et al.*, 2016). Molecular investigations are essential for deciphering the genetic causes of progeroid overlapping diseases. The hallmarks of progeroid syndromes include increased DNA damage accumulation, defective DNA repair, telomere dysfunction, aberrant nuclear architecture and chromatin structure, impaired cell cycle, senescence, disrupted epigenetics regulation, and lack of sWAT (Agarwal and Garg, 2006; Carrero **et al.*,* 2016; Niedernhofer *et al.*, 2018). 

*Cockayne Syndrome*

Cockayne Syndrome (CS) is a progressive rare autosomal recessive disorder, first described through the clinical study of two patients (Cockayne, 1936). This disease results in postnatal growth failure, and progressive neurologic dysfunction primarily due to demyelination, and photosensitivity (Nance and Berry, 1992). 

CS may manifest as delayed psychomotor development, behavioral and intellectual deterioration, microcephaly, increased or decreased muscle tone and reflexes, gait ataxia, tremor, incoordination, dysarthric speech, pigmentary degeneration of the retina, cataracts, optic atrophy or optic disk pallor, sensorineural hearing loss, dental complications, kidney complications, hyperinsulinemia or abnormal glucose tolerance, elevated serum cholesterol or lipoprotein levels, and very low levels of HDL-c (Nance and Berry, 1992).

The aged appearance may come from the expression of thin hair, diminished subcutaneous tissue, scaly skin, erythematous dermatitis on the dorsum of the hands and wrists, on the legs, and on the face and ears, worsened after exposure to the sun, small faces with sunken eyes and prominent superior maxillae (Cockayne, 1936).

*Xeroderma Pigmentosum*

Xeroderma Pigmentosum (XP) was first documented in 1884 when three affected patients were clinically studied, presenting freckle-like pigment spots which appeared simultaneously upon the face, neck, back of forearms, hands, upper arms, and legs below the knees (Crocker, 1884). Later, other studies showed that such cutaneous symptoms had a median age of onset of between one and two years, and about forty-five percent of the patients had basal cell carcinoma or squamous cell carcinoma of the skin. Many of them also presented neurologic abnormalities, including progressive mental deterioration, hyporeflexia or areflexia, and progressive deafness, associated with dwarfism and immature sexual development (Cleaver, 1968; Kraemer *et al.*, 1987). Next, James Cleaver discovered that fibroblasts obtained from XP patients displayed defective DNA repair after ultraviolet UV exposure (Cleaver, 1968). 

This condition has at least eight genetic groups, types A to G and a variant, which were identified through genetic complementation analysis (Tanaka, 1993). Cells from patients with the hereditary disease XP were expected to carry pathogenic variants in DNA repair genes. Their expression was either absent or much reduced compared to normal fibroblasts (Cleaver, 1968). This disorder presents over a 1,000-fold increased risk of skin cancer and a 10-fold increased risk of other tumors, along with progeroid symptoms. These symptoms were found in an XP patient, including an aged appearance, weight loss, epidermal atrophy, visual and hearing loss, ataxia, cerebral atrophy, hypertension, liver dysfunction, anemia, osteopenia, kyphosis, sarcopenia, and renal insufficiency (Niedernhofer *et al.*, 2006).

*Néstor-Guillermo Progeria Syndrome*

Néstor-Guillermo Progeria Syndrome (NGPS) is a chronic progeroid disease characterized by aging phenotypes, including growth retardation, thin limbs, and loss of sWAT. NGPS is caused by a homozygous pathogenic variant in the *BANF1* gene (c.34G>C; p.A12T), that encodes BANF1/BAF1 (barrier-to-autointegration factor 1) (Puente *et al.*, 2011). Two unrelated Spanish families were clinically investigated by Puente *et al.* (2011). Both had the c.34G>A [p.Ala12Thr] pathogenic variant in the *BANF1* gene. Skin fibroblasts from these patients exhibited deficient BANF1 levels and profound nuclear abnormalities, including blebs and aberrations. Concurrently, transfected mutant fibroblasts with an expression vector encoding an EGFP-BAF fusion protein, and confocal microscopy analysis, revealed that ectopic expression of EGFP-BAF in these progeroid fibroblasts rescued the nuclear abnormalities, confirming the causal role of the BAF p.Ala12Thr pathogenic variant (Puente **et al.*,* 2011). Later in the same year, Cabanillas *et al.* (2011) published a detailed clinical report of the two affected patients from the two unrelated families previously described.

Affected patients showed partial phenocopy of Hutchinson Gilford Progeria Syndrome (HGPS) and Mandibuloacral dysplasia (MAD) but without cardiovascular alterations and metabolic abnormalities. They presented a collection of clinical outcomes that suggested a new progeroid syndrome. Such manifestations included: very severe osteolysis with intense bone resorption, a long lifespan relative to HGPS and MAD, presence of eyebrows and eyelashes, and persistence of scalp hair. They also observed a generalized loss of sWAT over the limbs and trophic facial subcutaneous fat pad, abdomen, neck, and head, and dry and atrophic skin with small light-brown spots over the thorax, scalp, and limbs. Low levels of 25-OH-vitamin D and leptin were also seen (Cabanillas *et al.*, 2011; Puente *et al.*, 2011).

*Werner and Bloom Syndromes*

Werner (WS) and Bloom (BS) syndromes are rare recessive autosomal diseases characterized by clinical features of premature aging that are caused by loss-of-function pathogenic variants in the *WRN (RECQL2)* and *BLM (RECQL3)* genes, respectively (Ellis and German, 1996; Yu *et al.*, 1996; Hickson, 2003). WRN (WRN RecQ Like Helicase) and BLM (BLM RecQ Like Helicase) are ubiquitously expressed RECQ helicases that play major roles in a wide variety of DNA repair processes required for genomic integrity maintenance. WS was first described by Otto Werner in 1904, who presented the clinical WS phenotype as a “caricature of aging” (Werner 1985). WS patients exhibit metabolic complications including IR, DM, dyslipidemia, and fatty liver, as well as cataracts, cancer, and premature aging. Atherosclerosis is more frequent from the third decade onwards. At a molecular level, WS cells display a high rate of spontaneous mutations and karyotypic abnormalities, in addition to aberrant recombination, telomere defects, and hypersensitivity to DNA damage and/or cellular stress (Turaga *et al.*, 2009).

WS patients develop normally until the second decade of life, and the first clinical sign is the lack of peak pubertal growth. Between 20 and 30 years of age, patients begin to suffer from skin atrophy, gray hair, and hair loss. Soft tissue calcification is a feature often associated with ulcerations around the ankles (and occasionally elbows) that eventually may require lower limb amputation (Takemoto *et al.*, 2013). Other complications include type 2 DM, osteoporosis, bilateral ocular cataract, premature and severe forms of arteriosclerosis, peripheral neuropathy, and multiple cancers mainly perceived in middle age (Lauper *et al.*, 2013). These patients generally present a median age of death around 54 years, typically due to cancer or myocardial infarction (Goto, 1997; Goto **et al.*,* 2013; Martin *et al.*, 2021). WRN protein has exonuclease and helicase activities that are important for genome integrity maintenance. This protein interacts physically and functionally with enzymes that play central roles in DNA replication and repair. It is remarkable that replication and recombination functions also appear to underlie the telomeres maintenance by RecQ helicases (Turaga *et al.*, 2009).

BS, also referred to as congenital telangiectatic erythema, was first described in 1954 (Bloom, 1954). This progeroid syndrome is caused by pathogenic variants in the *BLM* gene that results in errors in the DNA replication process, and a pronounced number of chromosomal breaks and rearrangements, leading to the symptoms and clinical feature of BS (Bloom, 1954; Hickson, 2003). BS patients generally demonstrate postnatal growth retardation, facial butterfly rash, often after exposure to sunlight, defective cellular and humoral immunity, and an increased risk of cancer, besides a high prevalence of DM, dyslipidemia, and hepatic steatosis. Both WS and BS syndromes show metabolically phenocopies of lipodystrophy (reduction in sWAT) and obesity (Epstein *et al.*, 1966; Diaz *et al.*, 2006; Goh *et al.*, 2020).

*Hutchinson Gilford Progeria Syndrome*

HGPS is considered one of the most severe laminopathies, being included in the group of premature aging degenerative diseases. Patients live for an average of just 14.6 years, dying primarily due to myocardial infarction or strokes (Gordon *et al.*, 2014). HGPS was first described in 1886 by the British physician Jonathan Hutchinson and, later, by Hastings Gilford in 1904 (Hutchinson, 1886; McKusick, 2005). The main clinical manifestations of HGPS patients are sWAT loss, alopecia, Ca^2+^ dysfunction, vascular stiffening, delayed dentition, heart infarction, and progressive arteriosclerosis (Goldman *et al.*, 2004; Prokocimer *et al.*, 2013). Molecularly, HGPS patient cells have nuclear shape abnormalities, telomere shortening, genomic instability, alterations in epigenetic regulation and gene expression, mitochondrial dysfunction, and premature senescence. 

HGPS occurs due to the heterozygous silent pathogenic variant c.G608G in the *LMNA* gene (Eriksson *et al.*, 2003; De Sandre-Giovannoli *et al.*, 2003). *LMNA* encodes the prelamin-A, which undergoes post-translational processing, leading to transient production of different intermediates, including farnesylated prelamin-A and carboxymethylated prelamin-A (Lattanzi *et al.*, 2014). The zinc metalloproteinase STE24 homolog (ZMPSTE24) cleaves the prelamin-A in two independent steps: the first is the cleavage of the last three amino acids in the C-terminal region of farnesylated prelamin-A. This cleavage can also be performed by Ras converting CAAX endopeptidase 1 (RCE1). The second cleavage of farnesylated and carboxymethylated prelamin-A occurs at the leucine 647 (L647) and results in the removal of the last fifteen amino acids, producing the mature, unfarnesylated lamin-A (Lattanzi **et al.*,* 2014). The pathogenic variant c.G608G in the *LMNA* gene leads to the loss of the recognition site for the second cleavage of the farnesylated prelamin-A by ZMPSTE24 (Eriksson **et al.*,* 2003; De Sandre-Giovannoli **et al.*,* 2003). This change results in the accumulation of a permanently farnesylated and carboxymethylated dominant protein, referred to as progerin, disrupting the nuclear envelope (Broers **et al.*,* 2006; Bertrand *et al.*, 2011; Bidault *et al.*, 2020; Saxena and Kumar, 2020). Furthermore, the accumulation of farnesylated prelamin-A is related to nuclear enlargement, heterochromatin loss, euchromatin dispersion, and increased ROS production (Richards *et al.*, 2011).

*Type A Mandibuloacral Dysplasia with Lipodystrophy*

Type A Mandibuloacral Dysplasia with Lipodystrophy (MADA) is a rare autosomal recessive disease in which the patients commonly present slow and progressive osteolysis of the mandible, terminal phalanges, and clavicles, resulting in mandibular hypoplasia, dental crowding, and clavicular resorption, as well as skin abnormalities, acanthosis nigricans, and partial lipodystrophy. However, there is an absence of neurodegeneration. This condition is associated with accelerated aging and is usually identified after 4 or 5 years after birth (Novelli *et al.*, 2002). MADA patients express a partial lipodystrophy pattern of body fat distribution with degeneration of sWAT in the torso and limbs and accumulation in the face, neck, and trunks (Novelli **et al.*,* 2002). This syndrome may be associated with clinical features of metabolic syndromes, including IR, which was evidenced in the clinical study of three patients with MAD (Freidenberg *et al.*, 1992), impaired glucose tolerance, DM, and lack of breast development with regular or irregular menstrual periods in female patients (Cenni *et al.*, 2018). This disorder is caused by the accumulation of prelamin-A in MADA cells, leading to the restraint of cellular differentiation due to the impaired import of transcription factors required for adipogenic gene activation or stress response (Cenni **et al.*,* 2018). 

MAD was first reported by Young *et al.* (1971). Since then, other authors studied different cases of MAD in patients, such as Zina *et al.* (1981), Pallotta and Morgese (1984), and Tenconi *et al.* (1986), although the cause was still unknown. The official association between MADA and the *LMNA* gene was published in 2002, through the clinical and genetic investigation of five consanguineous Italian families, whose skin fibroblasts showed abnormal lamin nuclei (Novelli *et al.*, 2002).

Pathogenic variants in the *LMNA* gene, such as p.Arg471Cys, p.Arg527Cys, p.Arg527Leu, p.Arg527His, p.Ala529THR, p.Ala529Val, and p.Met540Ile (Marcelot *et al.*, 2020), cause the accumulation of prelamin A (non farnesylated) to toxic levels, along with the mutated prelamin A (farnesylated), affecting the whole organization of the nuclear envelope. The most common pathogenic variant responsible for the MADA phenotype is the homozygous missense substitution of c.1580G > A mapping in the exon 9 of the *LMNA* gene, resulting in the p.Arg527His mutated protein. These variants in the *LMNA* gene cause loss of interaction between lamin-A and other proteins, impacting stress recovery mechanisms in MADA cells, which means that repeated stress stimuli and failure to properly manage this condition led to senescence. These cells show nuclear dysmorphism, loss of peripheral heterochromatin, and nuclear lamina thickening (Cenni *et al.*, 2018).

*Type B Mandibuloacral Dysplasia with Lipodystrophy*

Type B Mandibuloacral Dysplasia with Lipodystrophy (MADB) is a rare autosomal recessive premature aging disease (Agarwal *et al.*, 2003a). MADB is characterized by IR, metabolic comorbidities, atrophic skin, brittle hair, generalized loss of sWAT, skeletal abnormalities such as mandibular and clavicular hypoplasia, and acro-osteolysis of the distal phalanges (Hitzert *et al.*, 2019). Although MADB and MADA have many similarities, MADB individuals develop early skeletal abnormalities (Agarwal **et al.*,* 2003a). *ZMPSTE24* pathogenic variants are responsible for many different diseases, depending on the degree of prelamin-A processing impairment (Shackleton *et al.*, 2005). MADB is caused by compound heterozygous or homozygous pathogenic variants in the *ZMPSTE24* gene, resulting in reduced activity of the metalloprotease ZMPSTE24. Compound heterozygous variants in the *ZMPSTE24* gene, such as p.Phe361fsX379/p.Trp340Arg (Agarwal **et al.*,* 2003a), p.Phe361fsX379/p.Asn265Ser (Shackleton **et al.*,* 2005; Agarwal **et al.*,* 2006), p.Gln41X/p.Pro248Leu (Miyoshi *et al.*, 2008), p.Tyr70fs/p.Asn265Ser (Cunningham *et al.*, 2010), and p.Pro248Leu/p.Trp450X (Ahmad *et al.*, 2010), as well as the homozygous variants p.Leu94Pro (Yaou *et al.*, 2011) and p.Tyr399Cys (Haye *et al.*, 2016), can partially or totally affect the functions of the metalloprotease ZMPSTE24, resulting in the accumulation of farnesylated prelamin-A and progressive loss of sWAT. *Zmpste24* ^*-/-*^ mice also displayed almost completed loss of sWAT due to the toxic accumulation of farnesylated prelamin-A (Bergo *et al.*, 2002; Pendás *et al.*, 2002).

*Wiedemann-Rautenstrauch Syndrome*

The *POLR3A* gene encodes the largest subunit of RNA polymerase III (Pol III), forming the catalytic core with POLR3B. Pol III is responsible for the transcription of different kinds of non-protein-coding RNAs, which regulate transcription, RNA processing, and translation (Sepehri and Hernandez 1997; Werner *et al.*, 2009; Wu *et al.*, 2021). This protein also acts in the proper function of the nucleolus, including ribosome assembly by enhancing 5S rRNA synthesis and protein translation, determining the metabolic state of the cell (Tiku and Antebi 2018; Báez-Becerra *et al.*, 2020).

Wiedemann-Rautenstrauch Syndrome (WRS) was first studied in 1977 (Rautenstrauch **et al.*,* 1977) and in 1979 (Wiedemann, 1979), both studies through clinical reports of patients with a progeroid syndrome, utilizing their lymphocytes and cultured skin fibroblasts. The relation between WRS and pathogenic variants in the *POLR3A* gene was confirmed by investigating DNA and RNA samples and fibroblast cultures of two affected Bulgarian families (Azmanov *et al.*, 2016), showing that the *POLR3A* gene is the primary locus for the WRS phenotype. Since then, studies have presented *POLR3A* biallelic variants that alter splicing and/or truncate translation and are associated with WRS, such as c.1909þ18G>A and c.2617C>T (Jay *et al.*, 2016), c.3337-5T>A, c.3337-11T>C, c.490+1G>A, c.2005C>T, c.760C>T, c.1572+1G>A, c.2617-1G>A, c.3G>T and c.*18C>T (Wambach *et al.*, 2018), all found in clinical and genetic analysis of WRS patients. Accordingly, these *POLR3A* alterations are the cause of the WRS progeroid disease.

WRS is sporadic and heterogeneous, characterized by intrauterine growth restriction (IUGR), poor postnatal weight gain, characteristic facial features, pseudohydrocephalus, generalized lipodystrophy, with an almost complete lack of subcutaneous fat and possible paradoxical caudal fat accumulation, premature alopecia, neonatal teeth, and teeth abnormalities (Rautenstrauch *et al.*, 1977; Wiedemann, 1979). The progressive generalized lipodystrophy manifests with local fatty tissue accumulations, and cachectic appearance (Paolacci *et al.*, 2017; Lessel and Kubisch, 2019).

*Ruijs-Aalfs Syndrome*

The *SPRTN* gene encodes to Spartan protein, a DNA-dependent metalloprotease associated with the replication machinery that repairs DNA-protein crosslinks (DPCs) through the SprT protease domain (Maskey *et al.*, 2014, 2017). DPCs derive from proteins covalently and irreversibly bound to DNA, such as Topoisomerase 1 (Top1), and the SPRTN (SprT-Like N-Terminal Domain) proteolytic activity, which upon DNA and ubiquitin-binding and promotes cleavage of DPC substrates and itself (Lopez-Mosqueda *et al.*, 2016; Li **et al.*,* 2019). Spartan malfunction, as a consequence of pathogenic variants such as c.721delA and c.350A>G (Lessel *et al.*, 2014), is responsible for replication stress, which has been suggested to cause DSBs, translocation mosaicism, and genomic instability. Thus, pathogenic variants in the *SPRTN* gene have been linked to cancer and aging, more specifically to the Ruijs-Aalfs syndrome (RJALS), an autosomal recessive disorder firstly described by Ruijs *et al.* (2003). RJALS individuals display genome instability, short stature, cataract, progeria, low body weight, micrognathia, triangular face, muscular atrophy, lipodystrophy, and early-onset hepatocellular carcinoma (Ruijs **et al.*,* 2003; Lessel *et al.*, 2014). 

The first association between *SPRTN* pathogenic variants, progeroid syndromes, and liver tumors was made in 2014, using *Sprtn* hypomorphic mice (Maskey *et al.*, 2014) and in primary skin fibroblasts, liver tumor biopsies, and lymphoblastoid cells (LCLs) from three progeroid patients, as well as in U2OS, and HEK293T cell lines (Lessel *et al.*, 2014). The pathogenic variants in the *SPRTN* gene, such as SPRTN-∆C and SPRTN-Y117C, and defects in DPC repair were shown in 2016 (Lopez-Mosqueda *et al.*, 2016; Stingele *et al.*, 2016; Vaz *et al.*, 2016).

## Genes related to DNA repair and genomic stability resulting in progeroid diseases with lipodystrophy

In the last years, a plethora of molecular findings unraveling the link between DNA damage/repair and adipogenesis in human and animal models has emerged. In this section, we will highlight the main findings concerning the role of genes related to DNA repair and genomic stability in progeroid syndromes with lipodystrophy. Table 2 summarizes the main findings of this section.

**Table 2 -:** The main progeroid and classical inherited lipodystrophies associated with changes in DNA repair and genome stability.

Gene	Protein	Cell model	Changes in redox homeostasis and DNA repair	Adipose tissue and metabolic commitments	Reference
*LMNA*	Lamin-A/C	Fibroblasts from FPLD2 subjects harboring the pathogenic variants in the *LMNA* genes (p.D47Y, p.L92F, p.L387V, p. R399H, p.L421P, and p. R482W)	Oxidative stress, mitochondrial dysfunction, cell cycle arrest, and premature senescence.	Partial loss of sWAT.	(Caron *et al.*, 2007)
*LMNA*	Lamin-A/C	Smooth Muscle Cells (SMCs) and fibroblasts from HGPS individuals carrying the pathogenic variant p.G608G.	PARP-1 suppression. Frequent DSBs, persistent activation of ATM and ATR. Higher levels of p-Chk-1, p-Chk-2, and p-p53. Mitochondrial dysfunction and NFE2L2 sequestration by progerin.	-	(Liu *et al.*, 2006; Zhang *et al.*, 2014; Kubben *et al.*, 2016)
*LMNA*	Lamin-A/C	Fibroblasts from MADA individuals harboring the pathogenic variant p.R527H in the *LMNA* gene.	High levels of chromosome aberrations. Increased phosphorylated ATM-S1981 foci and γ-H2AX, and p53 after IR treatment.	-	(Di Masi *et al.*, 2008)
*LMNA and ZMPSTE24*	Lamin-A/C and Zinc metalloproteinase STE24 homolog	*Zmpste24* ^*-/-*^ MEFs and HGPS fibroblasts carrying the pathogenic variant p.G608G.	High 53BP1 foci and increased protein levels of γH2AX and p-chk1. High sensibilization of *Zmpste24* ^*-/-*^ MEFs to DNA-damage agents. Delayed γH2AX/53BP1 co-localization. Higher DNA damage levels and decreased Rad51 foci formation.	-	(Liu *et al.*, 2005)
*ERCC8 and XPA*	CSA and XPA	CX (*Csa* ^*-/-*^ /Xpa^*-/-*^ ) mice with similar aspects of human progeria.	Increase in FAO/OXPHOS, decline of NAD^+^ and ATP levels, and increased levels of pAMPK due to PARP-1 activity.	Progressive loss of sWAT, low levels of TGs, and glucose. Decline in mature adipocyte size without crown-like (CL) structures, decreased sWAT and perigonadal WAT. Low levels of glucose, insulin, HOMA-IR, TGs, and leptin levels.	(Brace *et al.*, 2013; Brace **et al.*,* 2016)
*ERCC6* *and XPA*	CSB and XPA	*Csb* ^*m/m*^ /Xpa^*-/-*^ mice mimic the human progeroid CS syndrome.	Upregulation of genes associated with fatty acids synthesis and genes encoding antioxidant enzymes in the liver. Downregulation of genes involved in glycolysis, TCA, and OXPHOS.	sWAT loss and upregulation of *Lepr* and *Pparg*. Increased levels of TGs and glycogen accumulation. Low levels of glucose and IGF.	(Van Der Pluijm *et al.*, 2007)
*ERCC6*	CSB and XPC	*Csb* ^*m/m*^ /Xpc^*-/-*^ mice mimic the human progeroid CS syndrome.	-	sWAT loss.	(Van Der Pluijm *et al.*, 2007)
*ERCC1 and ERCC4*	Ercc1 and XPF	*Ercc1* ^*-/-*^ mice and XFE (XPF-ERCC1) fibroblasts from a subject harboring c.458 G>C in the *ERCC4* gene.	Sensitivity to oxidative stress. Upregulation of genes associated with fatty acids synthesis and genes encoding antioxidant enzymes in the liver.	sWAT loss and upregulation of Lepr and Pparg. Low levels of glucose, insulin, and IGF.	(Niedernhofer *et al.*, 2006)
*ERCC1*	ERCC1	*Ercc1* ^*-/-*^ fat depots from mice	Persistent DNA damage.	sWAT loss.	(Karakasilioti *et al.*, 2013)
*BANF1*	Barrier-to-autointegration factor	Skin fibroblasts from NGPS subjects harboring c.34 G>A (p.A12T) in the *BANF1* gene.	Defective PARP-1 activity and disrupted repair of oxidized DNA lesions.	Generalized lipodystrophy.	(Bolderson *et al.*, 2019)
*RECQL2*	DNA helicase, RECQ protein-like 2	*WRN* ^*-/-*^ human pluripotent stem cells (hPSCs) that were differentiated in adipocyte precursors (APs)	Reduced cell proliferation, shorter telomeres, and senescence.	Attenuated differentiation of *WRN* ^*-/-*^ APs to mature adipocytes and low adiponectin secretion. Low expression levels of *FABP4*, *CEBPA*, *GLUT4*, and *ADIPOQ* mRNAs in *WRN* ^*-/-*^ APs.	(Goh *et al.*, 2020)
*RECQL3*	DNA helicase, RECQ protein-like 3	*BLM* ^*-/-*^ human pluripotent stem cells (hPSCs) that were differentiated in adipocyte precursors	Reduced cell proliferation, shorter telomeres, and senescence.	Attenuated differentiation of *BLM* ^*-/-*^ APs to mature adipocytes. Low expression levels of *FABP4*, *CEBPA*, *GLUT4*, and *ADIPOQ* mRNAs in *WRN* ^*-/-*^ APs.	(Goh *et al.*, 2020)
*POLR3A*	RNA Polymerase III, subunit A	WRS fibroblasts carrying the pathogenic variant c.3772_3773delCT in the *POLR3A* gene.	Senescent cells. Increased levels of γH2AX and p53.	-	(Báez-Becerra *et al.*, 2020)
*SPRTN*	DNA-Dependent Metalloprotease Spartan	SPRTN-depleted U2OS cells, RJALS fibroblasts, *Sprtn* ^*F/-*^ MEFs, SPRTN-KO MEFs, and RJALS lymphoblastoid cells.	Increased accumulation of γH2AX and 53BP1 after CPT treatment in SPRTN-depleted U2OS cells transfected with the mutant p.Tyr117Cys or ∆C-TER SPRTN. Severe growth defect and increased levels of DSBs in patient fibroblasts. Increased numbers of 53BP1 nuclear bodies in *Sprtn* ^*F/-*^ MEFs. High sensitivity of lymphoblastoid cells derived from RJALS and SPRTN-KO MEFs to DPCs-inductor agents.	-	(Maskey *et al.*, 2014; Lessel *et al.*, 2014; Lopez-Mosqueda *et al.*, 2016)
*POLD1*	DNA polymerase delta 1	Individuals with MDPL harboring the heterozygous single codon deletion c.1812-1814delCTC (p.Ser605del) in the *POLD1* gene.	The catalytic subunit of POLD1 was affected, resulting in no detectable polymerase activity.	Progressive lack of sWAT in childhood, increased visceral adipose tissue (vWAT), and insulin resistance. Fibrosis in sWAT and high levels of genes from ECM, such as *TGFB1* and*FN1*.	(Shastry *et al.*, 2010; Weedon *et al.*, 2013; Reinier *et al.*, 2015; Okada *et al.*, 2017; Sasaki *et al.*, 2018; Wang *et al.*, 2018; Yu *et al.*, 2021)
*POLD1*	DNA polymerase delta 1	Individuals with MDPL harboring a heterozygous variant in the exonuclease domain of the *POLD1* gene (p.Arg507Cys)	-	Lipoatrophy in almost all body, but not in mechanic WAT.	(Pelosini *et al.*, 2014)
*POLD1*	DNA polymerase delta 1	Individuals with MDPL harboring heterozygous variants in the ZNF1 domain of the *POLD1* gene (c.3199 G>A; p.Glu1067Lys and c.3209 T>A; p.Ile1070Asn).	-	Generalized loss of sWAT and progeroid features in a patient harboring p.Ile1070Asn but not in the patients harboring p.Glu1067Lys, which had IR, elevated CK levels, and proteinuria.	(Ajluni *et al.*, 2017; Elouej *et al.*, 2017)
*POLD1*	DNA polymerase delta 1	HDFs from MDPL individuals harboring the heterozygous single codon deletion c.1812-1814delCTC (p.Ser605del) in the *POLD1* gene.	Nuclear envelope abnormalities, intranuclear accumulation of prelamin-A, high levels of micronuclei, cellular senescence, and growth decline. High levels of γH2AX foci after cisplatin-induced DSBs.	Diminished sWAT in limbs and increased AT in neck, abdomen, mesenteric regions, and retroperitoneal space. Progeroid features.	(Fiorillo *et al.*, 2018; Murdocca *et al.*, 2021)
*BSCL2*	Seipin	Leukocytes from CGL2 subjects harboring c.325dupA in the *BSCL2* gene.	High levels of oxidative stress and mitochondrial DNA damage. Upregulation of NFE2L2, APEX1, OGG1, and **α**-OGG1.	Severe loss of sWAT, Low HDL-c, low adiponectin and leptin levels, high levels of triglycerides in plasma	(Craveiro Sarmento *et al.*, 2020)
*CAV1* and *AGPAT2*	Caveolin-1 and 1-AGPAT 2	Whole blood from a subject harboring the heterozygous pathogenic variants c.479_480delTT (p.Phe160X) in the *CAV1* and c.51_52insGTC in the *AGPAT2* gene.	Downregulation of Fanconi anemia pathway, tricarboxylic acid (TCA) cycle, and oxidative phosphorylation (OXPHOS). Downregulation of *AGPAT2, RECQL4, and WRN* genes. Upregulation of the *ATM* gene.	Severe loss of sWAT. High levels of triglycerides in infancy. Low levels of caveolin-1 protein.	(Schrauwen *et al.*, 2015)

OMIM: Online Mendelian Inheritance in Man.

### The *LMNA* gene and FPLD2

The link between changes in redox homeostasis, cell cycle, and senescence was investigated in fibroblasts from FPLD2 subjects carrying the pathogenic variants p.D47Y, p.L92F, p.L387V, p.R399H, p.L421P, and p.R482W in the *LMNA* genes (Caron *et al.*, 2007). These pathogenic variants result in prelamin-A accumulation, the precursor of lamin-A, which was associated with the occurrence of mitochondrial dysfunction and higher levels of cytoplasmic ROS. Disturbances in the cell cycle and premature senescence were also found (Caron **et al.*,* 2007). Oxidative stress, inflammation, senescence, and calcification were also found in vascular smooth muscle cells (VSMCs) from FPLD2 subjects harboring R482W, D47Y, and R133L *LMNA* pathogenic variants (Afonso *et al.*, 2016). This study only investigated DSBs accumulation by evaluating the amount of γH2AX foci. Unrepaired DSBs accumulation was also verified in human coronary artery endothelial cells (HCAECs) transduced with adenoviral vectors containing Flag-tagged p.R482W prelamin-A cDNA It was also verified that pravastatin treatment decreased the levels of γH2AX foci (Bidault *et al.*, 2013).

Further, prelamin-A accumulation was directly associated with accumulation of DSBs in VSMCs infected with prelamin-A adenovirus (Liu *et al.*, 2013). The group performed microarray assays and found that DNA repair pathways responsible for the removal of DSBs were downregulated, suggesting that prelamin-A accumulation amplifies the DDR against DSBs. They also verified that the miRNA-141-3p levels were increased. This microRNA negatively regulates the ZMPSTE24, a prelamin-A maturation enzyme, which was considered a significant regulator of dysfunctional VSMCs from FPLD2 subjects. Although DNA repair pathways were not assessed in detail in this work, it is reasonable to suggest that the disrupted redox homeostasis found in those subjects could induce oxidized DNA damage and contribute to the pathophysiology of FPLD2. Indeed, Maynard and co-workers investigated the mechanism by which *Lmna* regulates the repair of oxidized DNA damage by the BER pathway in a mice model. They performed microarray gene expression and found that *Lmna* ^*-/-*^ MEFs (mouse embryonic fibroblasts) displayed an upregulation of genes related to the BER pathway and mitochondrial genome maintenance (Maynard **et al.*,* 2019). On the contrary, genes involved with metabolic processes and oxidative stress response mediated by NFE2L2 (nuclear factor erythroid 2-like 2; also termed NRF2) were downregulated. However, the authors did not explore the downregulated genes related to the metabolic process. Furthermore, they found that *Lmna* ^*-/-*^ MEFs were sensitive to DNA damage induced by hydrogen peroxide (H_2_O_2_) and menadione compared to *Lmna* ^*+/+*^ MEFs. Besides, the levels of 7,8-dihydro-8-oxoguanine (8-oxoG), the most abundant oxidized DNA level mainly repaired by the 8-oxoG DNA glycosylase (OGG1) from BER (Cadet *et al.*, 2003; Krokan and Bjørås, 2013), were higher in *Lmna* ^*-/-*^ MEFs relative to *Lmna* ^*+/+*^ MEFs after H_2_O_2_-induced DNA damage. These data indicate that this lesion is less efficiently repaired in the absence of *Lmna*, corroborating with results obtained by Comet assay, which revealed the repair efficiency of oxidized DNA lesions, including 8-oxodG and FapyG, was decreased in *Lmna* ^*-/-*^ MEFs relative to *Lmna* ^*+/+*^ MEFs. After H_2_O_2_-induced DNA damage, *Lmna* ^*-/-*^ MEFs also showed lower levels of *Parp-1*, *Lig3,* and *Polβ* mRNA expression as well as lower protein levels of PARP-1, LIG3, and Pol*β*. Interestingly, *Lmna* is required to APE1 and Polβ activities, which were PARP-1 dependent. *Lmna* depletion by siRNA also led to impaired BER in U2OS cells. Taken together, although these findings are very relevant to unravel the role of LMNA in the repair of oxidized DNA lesions, a link between BER and LMNA in the context of adipose tissue was not provided. 

### The *LMNA* gene and HGPS

Recent evidence revealed that accumulation of progerin causes defects in the expression and recruitment of DNA repair components, in addition to the suppression of Poly-ADP-ribose polymerase 1 (PARP-1) (Liu *et al.*, 2011; Zhang *et al.*, 2014). Zhang and co-workers found PARP-1 suppression in smooth muscle cells (SMCs) obtained from HGPS at protein levels and by immunofluorescence. This result was confirmed in HGPS fibroblasts carrying the pathogenic variant c.1824 C>T (p.G608G). Co-expression of PARP-1/GFP in SMCs revealed that progerin induces a mislocalization of a PARP-1 fraction to the cytosol (Zhang **et al.*,* 2014). PARP-1 usually plays a role in suppressing the NHEJ DNA repair mechanism and protecting HR (Broers **et al.*,* 2006; Bertrand *et al.*, 2011; Patel *et al.*, 2011; Zhang **et al.*,* 2014). Besides, most SMCs from HGPS individuals activated the error-prone NHEJ repair during S-phase, while HR was deficient during S-phase, leading to mitotic disaster and cell death (Zhang **et al.*,* 2014). These data indicate the role of progerin in regulating PARP-1 expression and NHEJ activity in SMCs from HGPS individuals.

The DDR to DSBs begins with the activation of ATM (Ataxia-Telangiectasia mutated) and ATR (ATM-and Rad3-related), which play central roles in DNA repair checkpoints. ATR is activated by broad DNA damage, whereas ATM is activated by DSBs. Activated ATM and ATR phosphorylate Chk-1 (Checkpoint kinase 1) and Chk-2 (Checkpoint kinase 2), initiating the signaling cascade that leads to p53 phosphorylation (Sancar *et al.*, 2004; Li and Zou, 2005). Liu et al compared aged HGPS fibroblasts harboring the pathogenic variant c.1824 C>T and normal BJ fibroblasts to determine whether DNA damage pathway checkpoints were persistently activated. In this study, it was observed that progeroid cells showed more frequent DSBs, and persistent activation of ATM and ATR checkpoints, which led to higher levels of phosphorylated Chk-1 and Chk-2 and, consequently, higher levels of phosphorylated p53 (Liu **et al.*,* 2006). 

Another study observed that although some DNA repair proteins, such as ATM, ATR, Chk1, Chk2, and p53 were activated, Rad50 and Rad51 were not recruited to the DNA damage regions (Liu *et al.*, 2008). Furthermore, surprisingly, XPA (Xeroderma pigmentosum complementation group A), a NER protein, was present in chromatin regions where DSBs had occurred in progeroid cells (Liu **et al.*,* 2008). The same was not observed in normal BJ fibroblasts, even when DSBs in DNA was induced by camptothecin (CPT). These findings suggest that the binding of XPA in DSBs regions prevents the recruitment of repair proteins such as Rad50 and Rad51 (Liu **et al.*,* 2008). In this way of thinking, XPA depletion was performed to verify whether the recruitment of repair proteins was restored. Indeed, a partial restoration of proteins such as Rad50, Rad51, and Ku70 was observed (Liu **et al.*,* 2008). 

Mitochondrial dysfunction and increased levels of ROS were also found in HGPS fibroblasts (Richards *et al.*, 2011). Accumulation of misrepaired DSBs and increased sensitivity to DNA damage agents, such as H_2_O_2_, were observed in HGPS fibroblasts. The treatment with N-acetyl cysteine (NAC), a ROS scavenger, decreased DSBs and improved cell growth (Richards **et al.*,* 2011). Besides, Kubben and co-workers found that although NFE2L2 (NRF2) protein levels did not change in HGPS fibroblasts, progerin sequesters NFE2L2 (NRF2), reducing its transcriptional activity since the sequestered NRF2 is mislocated to the nuclear periphery (Kubben **et al.*,* 2016). 

### The *LMNA* gene and MADA

To investigate the role of the *LMNA* R527H pathogenic variant in the cell cycle control and DDR, Alessandra di Masi and co-workers analyzed the response of MADA fibroblasts to DNA damage induced by IRa (Di Masi *et al.*, 2008). They found high levels of chromosome aberrations in G2-irradiated MADA fibroblasts, suggesting the occurrence of misrepaired DNA and that MADA cells are more sensitive to IRa than control fibroblasts. Basal levels of phosphorylated ATM (at S1981) were higher in MADA fibroblasts. Furthermore, increased phosphorylated ATM-S1981 foci were observed in almost 70% of MADA fibroblasts after X-ray treatment, suggesting accumulated DNA damage. Besides, as phosphorylation of γ-H2AX occurs around DSBs, being considered a marker for DSBs, immunofluorescence staining with the γ-H2AX antibody was performed. MADA cells presented a higher level of γ-H2AX after IRa treatment relative to control cells (Di Masi **et al.*,* 2008). Furthermore, p53 basal levels were 2-fold higher in MADA fibroblasts compared to control, suggesting that the prelamin-A accumulation in MADA cells can determine the persistence of misrepaired DNA damage. 

### The *ZMPSTE24* gene and MADB

The *ZMPSTE24* gene contribution to genomic stability and aging was also studied in models of progeroid phenotypes. Using *Zmpste24* ^*-/-*^ MEFs, Liu and co-workers discovered that the deficiency in *Zmpste24* resulted in cell cycle arrest and senescence. These cells also presented chromosomal instability and quickly accumulated DNA damage relative to controls (Liu **et al.*,* 2005). *Zmpste24* ^*-/-*^ MEFs had high 53BP1 foci and increased protein levels of γH2AX, a marker of DSBs, and phosphorylated chk1 (p-chk1), involved with DNA damage checkpoint response. They also found similar results in fibroblasts obtained from HGPS individuals. *Zmpste24* ^*-/-*^ MEFs also were sensitive to DNA-damage agents, such as those inducing DSBs [mitomycin (MMC), methylmethanesulfonate (MMS), CPT, and etoposide] and UV. After γ-irradiation, the number of γH2AX/53BP1 co-localized foci were delayed in *Zmpste24* ^*-/-*^ MEFs, suggesting that 53BP1 recruitment is affected. Besides, six and twelve hours after γ-irradiation, most of the 53BP1 foci disappeared in WT MEFs and fibroblasts. On the contrary, γH2AX/53BP1 co-localization was kept in *Zmpste24* ^*-/-*^ MEFs and HGPS fibroblasts, suggesting misrepaired DSBs. Later, they investigated whether defective DNA repair is associated with ZMPSTE24 deficiency. Using comet assay, the authors showed that *Zmpste24* ^*-/-*^ MEFs and HGPS fibroblasts had higher tail moment relative to controls, indicating that loss of *Zmpste24* and progerin compromised DNA repair. It was also suggested that DNA repair deficiency in *Zmpste24* ^*-/-*^ MEFs and HGPS fibroblasts may be due to decreased Rad51 foci formation. In another study, Varela and co-workers found that liver and heart from *Zmpste24* ^*-/-*^ mice displayed an upregulation of p53 target genes, such as *Gadd45a*, *p21* (*Cdkn1a*), and *Atf3*, as well as increased levels of γ-H2AX in the liver. *Zmpste24* deficiency also resulted in a senescent phenotype (Varela **et al.*,* 2005). Taken together, the authors revealed that the accumulation of farnesylated prelamin-A due to *Zmpste24* deficiency results in DNA damage accumulation, and the Rad51 recruitment is defective after γ-irradiation. 

### The *ERCC8 (CSA)*, *ERCC6 (CSB)*, and *XPA* genes and CS

Progressive loss of sWAT was observed in a model of CS mice (Brace *et al.*, 2013). CS is characterized by neurodegeneration, growth failure, and photosensitivity (Fousteri & Mullenders, 2008; Vessoni *et al.*, 2020). *Csa* ^*-/-*^ /Xpa^*-/-*^ (CX) mice showed more severe NER progeria, including small size and progressive loss of sWAT but not BAT. These mice also presented low levels of plasm triglycerides (TGs) and glucose. Therefore, the CX mice were a good model for studying human progeria. Later, the same group revealed changes in adiposity and lipid and glucose homeostasis in the CX mice model under chronic DNA damage induction, including IRa, crosslinking agent mitomycin (MMC), and ultraviolet (UV) radiation (Brace **et al.*,* 2016). They investigated how DNA damage affects energy metabolism and found that CX mice had a loss of sWAT and perigonadal WAT, as well as a decline in mature adipocyte size without inflammatory signals (crown-like - CL structures). Fasted CX mice had low glucose, insulin, HOMA-IR (homeostasis model assessment-estimated insulin resistance), and TGs in plasma compared to control mice. Circulating leptin levels were also decreased (Brace **et al.*,* 2016).

Another study also investigated the mitochondrial fatty acid oxidation (FAO) rate in these CX mice models. They found increased oxygen consumption rate (OCR), reduced respiratory exchange ratio (RER), as well as an upregulation of FAO-related genes in muscle from fasted CX mice (Brace *et al.*, 2016). They also verified the impact of DNA damage on FAO capacity. For this, they used mouse dermal fibroblasts (MDFs) isolated from tails of WT and CX mice, preadipocytes for CX mice, and human dermal fibroblasts (HDFs) from CSA and CSB patients. They confirmed an increase in FAO under UV-C treatment for the CX and CS models, as well as that MMC and IRa at high doses promoted a similar rise in FAO in CX MDFs, as they found for UV-C. These results suggested that increased FAO was a beneficial adaptive response to genotoxic stress induced by UV-C, MMC, and IRa and revealed a link between genotoxic stress and energy metabolism related to DNA damage. 

Furthermore, they showed that the ATP levels were decreased after UV-C or MMC treatments in WT MDFs and HDFs, which returned to normal levels almost 90 minutes later, indicating increased energy demands after the genotoxic stress induction. Interestingly, they also verified whether the ATP-reduced levels were linked to nicotinamide adenine dinucleotide (NAD^+^) depletion levels. NAD^+^ is a vital metabolite coenzyme for crucial metabolic pathways, such as glycolysis, TCA, and OXPHOS, as well as for ADP(ribosyl)ation reactions mediated by PARP-1 activity (Fouquerel and Sobol, 2014; Hurtado-Bagès *et al.*, 2020). They found a reduction in NAD^+^ levels in WT MDFs after both UV-C and MMC treatments, which is in accordance with ATP low levels. They also assessed PARP-1 activation through PAR accumulation to better understand whether the PARP-1 activity is associated with ATP and NAD^+^ depletion in WT and PARP-1 KO MDFs under genotoxic stress. They confirmed the occurrence of an increased PARylation in WT MDFs after two different genotoxic stresses (UV-C and MMC), but not in PARP-1 KO MDFs. In addition, they found that phosphorylated adenosine monophosphate (AMP)-activated protein kinase (pAMPK), which regulates metabolic changes due to ATP depletion, was also increased in a PARP-1 dependent manner in MDFs, and this result was confirmed in MDFs obtained from AMPK KO mice. Besides, CX mice showed low levels of NAD^+^ and increased levels of pAMPK in the liver. Altogether, these findings revealed that NAD^+^/ATP depletion and AMPK activation in cells/tissues from CX mice are dependent on PARP-1 and link different types of genotoxic stresses (UV-C, MMC, and IRa) to increased FAO. These data also reveal that CX mice are a model of chronic genotoxic stress and lipodystrophy due to congenital DNA repair deficiency. However, adiponectin, an important hormone produced by adipose tissue that activates AMPK phosphorylation and is reduced in congenital lipodystrophy (Antuna-Puente *et al.*, 2010; Lima *et al.*, 2016; Craveiro Sarmento *et al.*, 2020), was not investigated in this cell model.

Loss of sWAT was also observed in *Csb* ^*m/m*^ /Xpa^*-/-*^ mice that mimic the human progeroid CS syndrome (Van Der Pluijm *et al.*, 2007). These mice presented increased levels of TGs and glycogen accumulation and low serum glucose and IGF. Moreover, GH/IGF1 growth axis reduction was not due to reduced GH levels or pituitary abnormalities. Using transcriptome analysis, the authors found an upregulation of *Lepr* and *Pparg* genes that codify to the leptin receptor and peroxisome proliferator-activated receptor gamma, respectively. Furthermore, upregulation of genes associated with fatty acids synthesis and genes encoding antioxidant enzymes in the liver from *Csb* ^*m/m*^ /Xpa^*-/-*^ mice were found. In contrast, genes involved in glycolysis, TCA, OXPHOS, and controlling growth (*Igf1*) were downregulated. A similar loss of sWAT was similarly found in *Csb* ^*m/m*^ /Xpc^*-/-*^ mice. The authors also compared the *Csb* ^*m/m*^ /Xpa^*-/-*^ mice model with naturally aged mice. They found that the latter also presented accumulation of glycogen and TGs, and repression of genes related to oxidative metabolism and the IGF axis (Van Der Pluijm **et al.*,* 2007).

Kamenisch and co-workers revealed that the presence of CSA and CSB proteins in mitochondria are essential for protecting against loss of sWAT (Kamenisch **et al.*,* 2010). After H_2_O_2_ treatment, oxidatively stressed WT fibroblasts had detectable levels of CSA and CSB within mitochondria. Further, they detected interactions between CSA or CSB and mitochondrial OGG1 (mtOGG1) and single-stranded DNA binding protein (mtSSBP1) only in H_2_O_2_-stressed WT cells. Cells from CSA and CSB patients and sWAT from *Csb* ^*m/m*^ and *Csa* ^*-/-*^ mice showed higher levels of mutations in mtDNA that was age-dependent. Fat tissue from 130-weak-old *Csb* ^*m/m*^ mice had a higher accumulation of mtDNA mutations. They also investigated whether the reduction of sWAT in *Csb* ^*m/m*^ mice was due to a reduction in the fat cell size or number. They found that sWAT from 130-weak-old *Csb* ^*m/m*^ mice had higher levels of macrophages containing granular lipofuscin in lysosomes, a phagocytosis marker, suggesting that the loss of sWAT in *Csb* ^*m/m*^ and *Csa* ^*-/-*^ mice is mediated on the fat number (Kamenisch **et al.*,* 2010). However, the authors did not investigate the metabolic parameters nor the levels of antioxidant adipokines, such as adiponectin, in *Csb* ^*m/m*^ and *Csa* ^*-/-*^ mice. It is known that mitochondrial function is crucial for adiponectin synthesis in adipocytes (Eun *et al.*, 2007), adiponectin is downregulated in lipodystrophies (Antuna-Puente *et al.*, 2010), and this adipose tissue-produced hormone induces antioxidant responses through NRF2 activation (Li *et al.*, 2015; Ren *et al.*, 2017). However, whether adiponectin is involved with the maintenance of mtDNA homeostasis in lipodystrophies remains to be shown.

### The *ERCC4 (XPF)* and *ERCC1* genes and XP

Another association between DNA repair deficiency, absence of adipose tissue, and aging was also found (Niedernhofer *et al.*, 2006). The authors used the *Ercc1* ^*-/-*^ mice model as an accurate model of an XPF-ERCC1 (XFE) progeroid patient. They found that *Ercc1* ^*-/-*^ mice presented weight loss, and the primary mouse embryonic fibroblasts isolated from these mice were sensitive to oxidative stress induced by treatment with H_2_O_2_ and paraquat. They showed premature aging in several organs and had liver failure. As in the *Csb* ^*m/m*^ /Xpa^*-/-*^ mice model, a transcriptomic analysis from *Ercc1* ^*-/-*^ mice liver revealed an upregulation of genes associated with fatty acids synthesis and genes encoding antioxidant enzymes. Furthermore, *Lepr* and *Pparg* genes were upregulated, and the Adipor2 (adiponectin receptor 2) was downregulated. On the contrary, low levels of glucose and IGF were also found in this cell model. Taken together, these findings show that both models of NER progeria are associated with loss of adipose tissue homeostasis, and this can be due to the accumulation of ROS and DNA damage accumulation. This results in the downregulation of GH/IGF1 hormonal axis in *Ercc1* ^*-/-*^ mice to moderate the metabolism, indicating that IGF1 reduction may have beneficial effects in extending lifespan in mice. However, since DNA damage accumulates, degenerative processes will occur, such as loss of sWAT, resulting in aging. CS patients have been previously reported with low levels of IGF1 serum and decreased fat deposition (László and Simon, 1986; Park *et al.*, 1994). As observed in *Csb* ^*m/m*^ /Xpa^*-/-*^ mice model, the reduction of genes related to the GH/IGF1 growth axis in Ercc1^-/-^ mice liver was also not due to reduced GH levels or pituitary abnormalities.

In the same way, Karakasilioti and co-workers provided evidence for a causal link between persistent DNA damage and the gradual appearance of progressive lipodystrophy in NER progeria (Karakasilioti **et al.*,* 2013). To increase the understanding of the role of unrepaired DNA damage in adipose tissue degeneration, they found that DNA damage signaling resulted in fat depletion due to chronic inflammation in *Ercc1* ^*-/-*^ fat depots from mice or in adipocytes (Karakasilioti **et al.*,* 2013). These mice presented a gradual reduction of epididymal WAT (eWAT), cervical, interscapular, and sWAT depots. To distinguish primary and secondary mechanisms related to fat depletion in Ercc1-deficient mice, the authors also created *aP2-Ercc1* ^*F/-*^ mice, which present aP2 expression mainly but not exclusively in mature adipocytes (Shan *et al.*, 2013), while *Ercc1* is later deleted. This strategy aims to verify the effect of time-dependent accumulation of DNA damage only on adult AT depots. Progressive lipodystrophy was also found in eWAT, interscapular, and sWAT from *aP2-Ercc1* ^*F/-*^ mice, which had high TGs and low levels of adiponectin. They also had decreased interscapular BAT depots. 

To further understand the role of ERCC1 in WAT, the authors analyzed the transcriptome of eWAT depots and found more than 2.000 differentially expressed genes. Genes related to response to DSBs (for ex. ATM signaling), response to stress (for ex. NRF2-related oxidative stress response), nuclear receptor (for ex. PPAR), and pro-inflammatory (TNF, NFκB) signaling were upregulated. Accumulation of γ-H2AX, phosphorylated ATM (pATM), RAD51, and FANCI was observed in adipocytes from *aP2-Ercc1* ^*F/-*^ mice. Ablation of Ercc1 also triggered a gradual accumulation of persistent DNA damage, resulting in adipocytes’ necrosis. 

### The *BANF1* gene and NGPS

**Figure 1 -:**
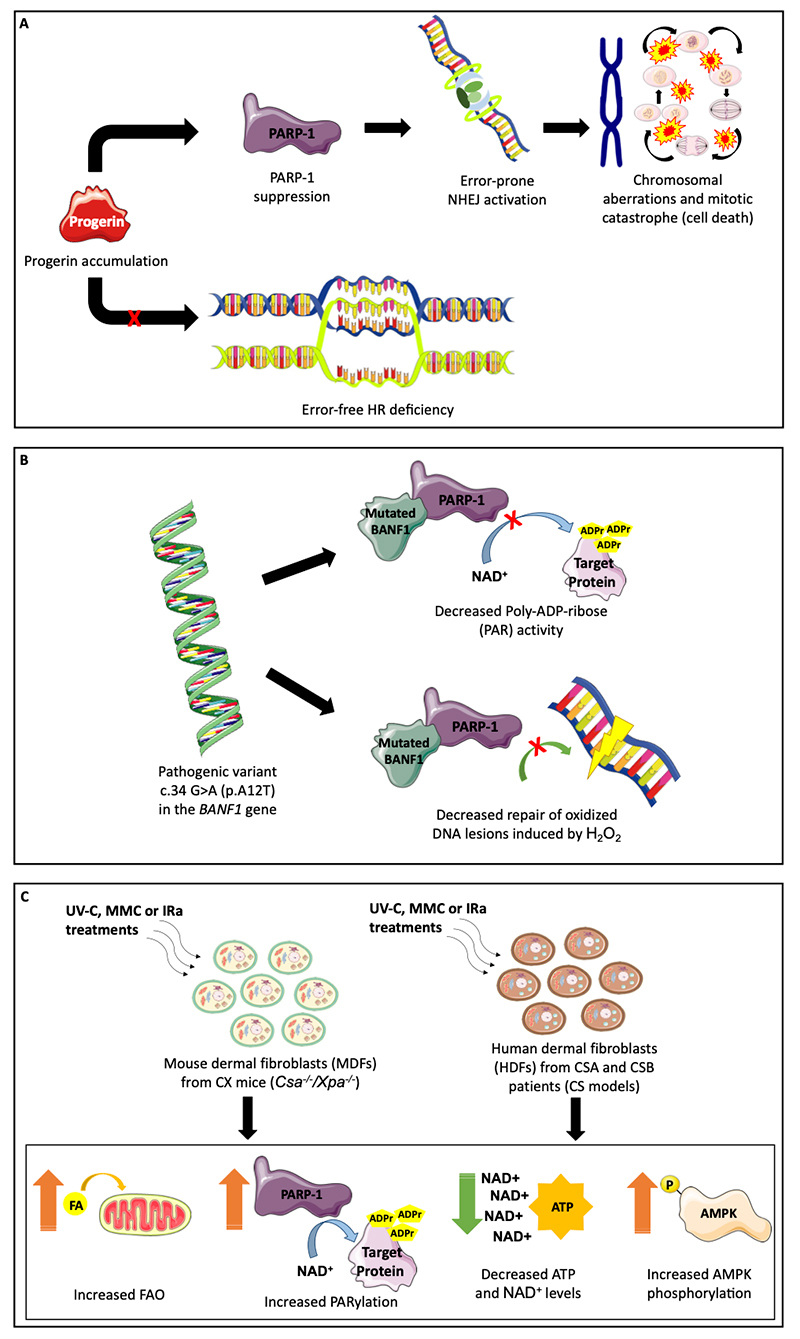
Modulation of PARP-1 activity in HGPS, NGPS, and CS. (A) In smooth muscle cells (SMCs) and fibroblasts from HGPS individuals, progerin accumulation results in suppression of PARP-1 protein levels. SMCs activated the error-prone NHEJ repair during S-phase, while HR was deficient during S-phase, leading to mitotic disaster and cell death (Zhang *et al.*, 2014). (B) Following H_2_O_2_-induced oxidative stress in HEK293T cells, the mutated BANF1 protein interacts with the NAD^+^-binding domain of PARP-1, directly regulating its ADR-ribose (ADPr) activity. Furthermore, NGPS fibroblasts showed decreased PARylation and repair of H_2_O_2_-induced DNA lesions (Bolderson *et al.*, 2019). (C) In CX mice liver, increased FAO, low levels of NAD^+^, and increased levels of pAMPK were found. These findings revealed that NAD^+^/ATP depletion and AMPK activation in cells/tissues from CX mice are dependent on PARP-1 (Brace **et al.*,* 2012, 2016). Pieces of this image are from the SMART website (Les Laboratoires Servier).

Barrier-to-autointegration factor 1 (BANF1) is another protein related to severe premature aging and DNA damage/repair in NGPS (Bolderson *et al.*, 2019; Rose *et al.*, 2021). This protein is essential for controlling the DDR against oxidative stress by regulating PARP-1 activity (Bolderson **et al.*,* 2019). The authors found that skin fibroblasts from NGPS subjects harboring the c.34 G>A (p.A12T) pathogenic variant in the *BANF1* gene had decreased PARP-1 poly-ADP-ribose activity and repair of oxidized DNA lesions induced by H_2_O_2_. Biochemical experiments in HEK293T cells revealed that the mutated BANF1 protein directly inhibits PARP-1 activity by binding to its NAD^+^ binding domain, maintaining the cellular levels of NAD^+^ after DNA damage induction. They concluded that the subcellular levels of the BANF1 protein are critical to reset PARP-1 activity under oxidative stress conditions, and the accumulation of oxidized DNA damage is associated with HGPS development. Figure 1 shows the main molecular findings concerning PARP-1 activity in different cellular models of progeroid lipodystrophy (HGPS, NGPS, and CS).

### The *POLD1* gene and MDPL

A multisystem disease characterized by mandibular hypoplasia, deafness, progeroid features, and lipodystrophy (MDPL) was associated with pathogenic variants in the *POLD1* gene in seven patients (Shastry *et al.*, 2010). Two MDPL patients from this work (named 300.4 and 500.4) were also described by Shastry and co-workers (named P3 and P4) (Weedon *et al.*, 2013) (Shastry **et al.*,* 2010). Shastry and co-workers found a progressive loss of sWAT with partial lipodystrophy in four young adults, while generalized lipodystrophy was confirmed only in older patients. Weedon and co-workers found that, although the patients presented normal body weight and appearance at birth, they had a lack of sWAT in early childhood. Loss of sWAT in adulthood was observed in almost all sites, which contrasted with a remarkable increase of vWAT, resulting in a greater ratio of vWAT to sWAT (Weedon **et al.*,* 2013). They also presented IR, fibrosis of sWAT, and increased levels of fundamental extracellular matrix (ECM) genes, such as transforming growth factor (TGF)-β (*TGFB1*) and fibronectin (*FN1*) (Weedon **et al.*,* 2013). They identified an in-frame deletion c.1812-1814delCTC (p.Ser605del) in the *POLD1* gene in two patients, which affects the polymerase’s active site. Assays for measuring the polymerase and exonuclease activities revealed that the heterozygous in-frame deletion affected the polymerase activity, which was not detectable, whereas the exonuclease activity was decreased. Another study reported a novel pathogenic variant in the exonuclease domain of the *POLD1* gene (p.Arg507Cys). However, they did not perform functional experiments to characterize better how the activities of POLD1 are affected. In this case, the MDPL patient also had a loss of sWAT nearly in the entire body, except for mechanical adipose tissue (Pelosini *et al.*, 2014). Reinier and co-workers also described a patient harboring the c.1812-1814delCTC (p.Ser605del) pathogenic variant in the POLD1 gene who had severe lipodystrophy and progeroid features (Reinier **et al.*,* 2015). The exact pathogenic variant was also found in Japanese subjects for two independent groups, suggesting that c.1812-1814delCTC (p.Ser605del) is a deletion hot spot variant associated with MDPL (Okada *et al.*, 2017; Sasaki *et al.*, 2018). Wang and co-workers reported the same family with subjects harboring two rare progeroid diseases, WS and MDPL (Wang **et al.*,* 2018). The proband had the hot spot c.1812-1814delCTC (p.Ser605del) pathogenic variant in the *POLD1* gene. He presented a progressive loss of sWAT and progeroid features that started at 18 months. His three brothers who had WS showed a heterozygous frameshift pathogenic variant in the *WRN* gene (c.919_923delACTGA, p.Thr307ThrfsX5) (Wang **et al.*,* 2018). Another MDPL case due to the hot spot heterozygous in-frame deletion was also described in a Chinese patient who presented progressive loss of sWAT that started at the age of seven (Yu *et al.*, 2021).

Elouej and co-workers described a new heterozygous pathogenic variant affecting the zinc finger 2 (ZNF2) domain in the *POLD1* gene (c.3209 T>A; p.Ile1070Asn) (Elouej **et al.*,* 2017). The patient developed lipodystrophy and progeroid facial features. Predictions using the PredictProtein server suggested that the substitution of isoleucine by asparagine at position 1070 can disrupt the Fe-S cluster within the CysB motif from the ZNF domain. Furthermore, Ajluni and co-workers also reported a new pathogenic variant affecting the ZNF2 domain (c.3199 G>A; p.Glu1067Lys). However, in this case, the two related subjects had reduced sWAT in the extremities but not around the neck, face, and abdominal wall. They presented IR, elevated CK levels, and proteinuria. They did not show progeroid features and deafness. In addition, while the MDPL patient had a high amount of nuclear atypia and disorganization in liver biopsy samples, these changes in the nuclear envelope integrity were lower when compared to patients harboring *LMNA*-pathogenic variants (p.R60G, p.R482Q, and p.R349W) (Ajluni **et al.*,* 2017).

Mechanistically, two independent works found that the progeroid features of two MDPL patients harboring the in-frame heterozygous deletion p.Ser605del are related to impaired DNA repair capacity (Fiorillo *et al.*, 2018; Murdocca *et al.*, 2021). Fiorillo and coworkers found that an MDPL patient carrying the heterozygous single codon deletion c.1812-1814delCTC (p.Ser605del) in the *POLD1* gene showed type 2 diabetes, hyperinsulinemia, and IR. HDFs obtained from this patient had nuclear envelope abnormalities, intranuclear accumulation of prelamin-A, high levels of micronuclei, cellular senescence, and growth decline. The authors studied the link between MDPL and DNA damage accumulation. After cisplatin-induced DSBs, they found high levels of γH2AX foci and a DNA repair recovery delay in HDFs compared with WT HDFs (Fiorillo **et al.*,* 2018). Similar results were found in HDFs obtained from a second MDPL patient (Murdocca* *et al.*,* 2021).

Although all these findings ratified the role of POLD1 in adipose tissue homeostasis, our understanding of how these pathogenic variants result in cellular defects in adipose tissue is scarce, and the mechanisms that link disrupted POLD1 activity to different diseases need to be further clarified. 

### The *RECQL2 (WRN)* and *RECQL3 (BLM)* genes and WS and BS

WS and BS have been studied as a model for deciphering adipose tissue senescence. Using CRISPR/Cas9, Goh and co-workers generated *WRN* ^*-/-*^ and *BLM* ^*-/-*^ human pluripotent stem cells (hPSCs), which were differentiated in adipocyte precursors (APs) (Goh **et al.*,* 2020). They found that *WRN* ^*-/-*^ and *BLM* ^*-/-*^ APs displayed reduced cell proliferation, shorter telomeres, and senescence. The latter was confirmed by measuring the mRNA levels of the senescent biomarkers: *p16*, *p21*, *Activin A*, *IL-6*, and *IL-8*. These findings suggest that preadipocyte senescence may be the cause of metabolic complications in WS and BS. In another study, Turaga and co-workers transfected human diploid fibroblasts with a siRNA against *WRN* mRNA, which became senescent and presented a similar gene expression profile relative to fibroblasts established from old donor patients (Turaga **et al.*,* 2009). From 660 differentially expressed genes found in the microarray analysis, 542 (82%) were downregulated, whereas 118 genes (18%) were upregulated, revealing a repression scenario in cells with lower *WRN* levels. Western blotting was performed for fourteen proteins and they confirmed the downregulation of: CCNB1 (Cyclin B1), CDC2 (Cyclin-dependent kinase 1), FANCD2 (Fanconi anemia complementation group D2), FANCI (Fanconi anemia complementation group I), FANCJ (Fanconi anemia complementation group J), FAS (Fas cell surface death receptor), HUWE1 (E3 ubiquitin-protein ligase), MRE11A (Meiotic Recombination 11 homolog A), KIF4A (Kinesin family member 4A), LMNA (Lamin A/C), MAPK8 (Mitogen-activated protein kinase 8), POLD1 (DNA polymerase δ subunit 1), SAFB1 (Scaffold attachment factor B1), and TOP2A (Topoisomerase II alpha). The gene set enrichment analysis revealed that the genes related to adipocyte differentiation were downregulated in *WRN*-knockdown fibroblasts (Turaga **et al.*,* 2009). To confirm this observation, the authors also transfected the 3T3-L1 mice preadipocytes with a siRNA against *Wrn* mRNA. The expression of adipogenic markers, such as C/EBPβ (CCAAT/enhancer binding protein β) and fatty acid synthase (FASN), was decreased. These data link the role of WRN and BLM proteins in the maintenance of adipose tissue homeostasis.

### The *POLR3A* gene and WRS

The *POLR3A* gene is crucial for cell function and metabolism. Pathogenic variants can alter its ability to interact with DNA, causing drastic changes in its transcriptional function and RNA polymerase I and II regulation. This scenario is associated with an early senescent phenotype found in primary WRS fibroblasts carrying the pathogenic variant c.3772_3773delCT (p.Leu1258Glyfs*12) in the *POLR3A* gene. WRS fibroblasts presented increased expression levels of the mutant POLR3A protein in the nucleoplasm, which was not expressed in control fibroblasts. Senescence was revealed by the presence of higher beta-galactosidase-positive WRS cells and increased levels of p16 protein expression. Decreased telomere length, increased DNA damage, and variations in the morphology and number of nucleolus were also seen (Báez-Becerra *et al.*, 2020). WRS fibroblasts exhibited strong phosphorylation levels of H2X in the Ser139 (termed **γ**H2AX**)** and p53 (in the Ser15) relative to control cells, which were associated with increased nuclear staining. These results indicate that WRS fibroblasts show an increase in DNA damage that can induce DDR and, consequently, a p53-mediated cell senescence. Also, a pathway of POLR3-mediated p53 regulation is likely lost upon *POLR3A* pathogenic variants in WRS fibroblasts. Altogether, these results revealed a link between *POLR3A* variants and DDR in WRS fibroblasts.

*The SPRTN gene and RJALS*

Lessel *et al.* (2014), proposed a clinical study of three patients with early-onset hepatocellular carcinoma (HCC), genomic instability, and progeroid features. To analyze Spartan function in DNA damage, U2OS cells were depleted of endogenous SPRTN using siRNA. Later, these SPRTN knockdown cells were transfected with the WT SPRTN, the mutant p.Tyr117Cys SPRTN, or ∆C-TER SPRTN. The authors found that the WT and mutated p.Tyr117Cys SPRTN formed nuclear foci, but not the mutated ∆C-TER SPRTN. The histological and immunohistochemical investigation of the patients’ liver tumor biopsies showed an increased accumulation of γH2AX and 53BP1 after CPT treatment, a chemotherapeutic agent that induces DPCs, including Top1 cleavage complex (Top1ccs). This result was also confirmed in SPRTN-knockdown U2OS cells expressing the mutant p.Tyr117Cys SPRTN and ∆C-TER SPRTN. Severe growth defects were also observed in patient fibroblasts, which showed increased levels of DSBs when in the S-phase. Indeed, transfection of patient fibroblasts with WT SPRTN efficiently corrected the replication defects and reestablished cellular proliferation. These results revealed that cells expressing mutant SPRTN were unable to recover DNA replication fork progression, leading to DNA replication stress and replication-related DNA damage, especially DSBs (Lessel **et al.*,* 2014). In the same year, Maskey *et al.* (2014) demonstrated that γH2AX foci, a marker of DNA damage, were markedly increased in *Sprtn* ^*F/-*^ MEFs after 4-hydroxytamoxifen (4-OHT) treatments, and that *Sprtn* ^*-/-*^ MEFs had increased numbers of 53BP1 nuclear bodies, indicating incomplete DNA replication.

To better characterize the molecular mechanism by which SPRTN contributes to genomic stability, Lopez-Mosqueda *et al.* (2016) verified the role of SPRTN in resolving DPCs. They found that SPRTN-KO MEFs were sensitive to agents that induce DPCs, such as formaldehyde, etoposide, and CPT. Also, B-II-1 lymphoblastoid cells derived from RJALS were sensitive to those DPCs-inductor agents. These cells also exhibited more γ-H2AX staining after formaldehyde and etoposide treatments (Lopez-Mosqueda **et al.*,* 2016). They also confirmed that SPRTN is a DNA binding protease involved with the removal of DPCs *in vivo* and in *vitro*. These data are consistent with accelerated aging phenotypes observed in the hypomorphic *SPRTN* mouse model, linking DPC repair deficiency to segmental progeroid syndrome (Lopez-Mosqueda **et al.*,* 2016).

Vaz *et al.* (2016) confirmed that SPRTN protease is a protein specialized in the repair of DPCs, being essential for DNA replication progression and genome stability. They found that RJALS patient cells and SPRTN-depleted cells were hypersensitive to agents inducing DPCs. Besides, HeLa cells transfected with ∆-SPRTN showed a higher average number of 53BP1 foci relative to controls after CPT treatment. This was observed only in cyclin A-positive ∆-SPRTN HeLa cells, suggesting a role of SPRTN in preventing DSBs induced by DPCs during the S-phase. Thus, RJALS cells are unable to process DPCs during DNA replication, leading to DNA replication stress, one of the main causes of genome instability and cancer (Vaz **et al.*,* 2016).

Maskey *et al.* (2017) used *Sprtn* hypomorphic MEFs, which express reduced levels of Spartan but have a normal cell-cycle distribution, to verify the role of Spartan in the repair of Top1ccs, a bulky CPT-induced DPC that blocks replication forks. They found that *Sprtn* hypomorphic MEFs exhibited high CPT sensitivity compared to control MEFs, suggesting that Spartan may play a role in Top1ccs repair. Furthermore, they studied the effects of DPCs in *Sprtn* hypomorphic mice, which recapitulate phenotypes observed on RJALS. They found an accumulation of Top1ccs in the liver, indicating an increased binding of Top1 to DNA (Maskey **et al.*,* 2017). Therefore, given that Spartan plays a significant role in DNA stability by being responsible for DPC repair throughout DNA replication, pathogenic variants in the *SPRTN* gene affect DNA repair and are associated with hepatocellular carcinoma and premature aging, such as in RJALS. 

**Figure 2 -:**
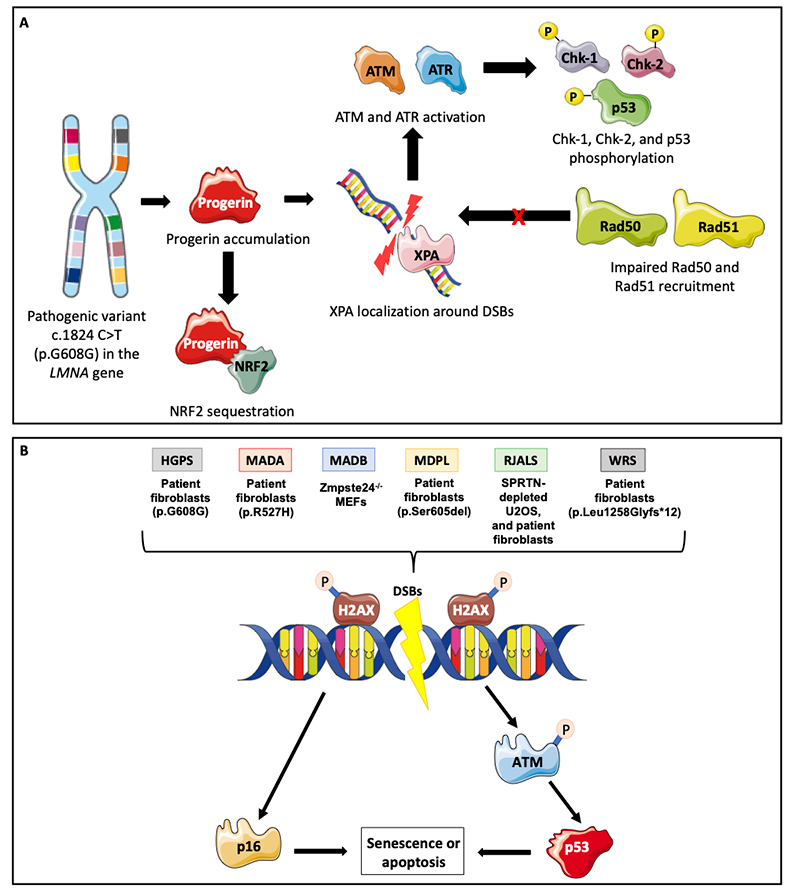
The main DNA repair changes in progeroid diseases with remarkable loss of sWAT depicting the occurrence of unrepaired DSBs and persistent γ-H2AX. (A) In HGPS, progerin accumulation results in frequent DSBs, phosphorylation of ATM, ATR, Chk1, Chk2, and p53 (Liu *et al.*, 2006). XPA binding to or near DSBs impairs Rad50 and Rad51 recruitment to damaged DNA (Liu **et al.*,* 2008). Furthermore, accumulated progerin sequesters NRF2, attenuating its transcriptional activity (Kubben *et al.*, 2016). (B) Increased levels of DSBs are also found in HGPS, MADA, MADB, MDPL, RJALS, and WRS, revealing a common DDR activation by phosphorylation of ATM and p53 (Liu **et al.*,* 2005; Di Masi *et al.*, 2008; Maskey *et al.*, 2014; Lessel *et al.*, 2014; Fiorillo *et al.*, 2018; Báez-Becerra *et al.*, 2020; Murdocca *et al.*, 2021). This mechanism is involved with senescence and apoptosis (Smith *et al.*, 2021). Pieces of this image are from the SMART website (Les Laboratoires Servier).

Figure 2 shows a model depicting the occurrence of unrepaired DSBs and persistent γ-H2AX in some progeroid diseases with remarkable loss of sWAT. As reviewed here, activation of DDR in HGPS, MADA, MADB, WRS, RJALS, and MDPL was seen, revealing an association among DSBs’ accumulation, aging, and loss of sWAT. Indeed, the role of p53 in the maintenance of sWAT homeostasis during aging was confirmed by Liu and co-workers (Liu *et al.*, 2018). Using adipocyte-specific MDM2-knockout mice (Adipo-MDM2-KO), the authors found that *MDM2* mRNA and protein levels are selectively downregulated in sWAT and BAT, while p53 and p21 were induced in both AT depots. Adipose senescence and apoptosis were observed in aged adipose tissue, and adipocytes had an aberrant expression of pro-inflammatory cytokines, such as TNFα and IL-6, while the p21 senescent marker was increased. Furthermore, adipocytes from old Adipo-MDM2-KO showed remarkable and progressive loss of SWAT, eWAT, and BAT, and leptin and adiponectin levels were nearly undetectable, revealing an early onset of lipodystrophy in this mice model. These mice also had diabetes, fatty liver, and higher levels of TGs, insulin, and glucose in plasma. The role of p53 in adipocytes’ homeostasis was validated by the generation of a DKO mice model lacking p53. DKO mice showed a rescued phenotype of sWAT loss and improvement of the metabolic parameters, confirming that the p53 activation is related to the MDM2-null phenotypes. However, the contribution of DNA damage/repair to the MDM2-p53 axis in the Adipo-MDM2-KO mice model was not assessed.

## Adipose tissue-related genes associated with changes in the expression of dna repair and Oxidative stress genes

### The *BSCL2* gene and CGL2

The ER-localized seipin, an adipose tissue-related protein involved with LDs assembly (Wang *et al.*, 2016), was associated with changes in redox homeostasis (Craveiro Sarmento *et al.*, 2020). The authors verified that blood leukocytes from CGL2 individuals carrying the pathogenic variant c.325dupA (p.T109Nfs*5) in the *BSCL2* gene displayed higher levels of serum oxidized glutathione and malondialdehyde, indicating the occurrence of oxidative stress and lipid peroxidation on blood from individuals presenting a paucity of sWAT since birth. Using LX-PCR to quantify the levels of mitochondrial DNA (mtDNA) damage, they found that the number of mtDNA lesions obtained from blood leukocytes from CGL2 subjects was higher relative to the control groups. Besides, the levels of mtDNA lesions were positively correlated with *NFE2L2* (*NRF2*) mRNA levels, suggesting the activation of NRF2 antioxidant responses. A positive correlation was also found between *NRF2* mRNA and serum adiponectin levels. Even in low levels in CGL2 subjects, this finding suggests that NRF2 activation occurred in an adiponectin-dependent manner. More studies are needed to unravel the relationship between NRF2 and adiponectin in the context of loss of sWAT.

Moreover, mitochondrial bioinformatics predictions by Mitochondrial Disease Database (MITODB) (Scheibye-Knudsen *et al.*, 2013), a software that determines whether a disease could be associated with mitochondrial commitments according to its phenotypes, revealed that CGL2 has a high probability (mito-score 92) of being related to mitochondrial disturbs since its clinical spectrum includes lipodystrophy, hepatomegaly, HTG, muscle hypertrophy, muscle hyperplasia, hypertrophic cardiomyopathy, and bone cysts (Lima *et al.*, 2016). These findings are in accordance with recently published data (Combot *et al.*, 2022), who found that seipin is localized at ER-mitochondria sites and has a role in the Ca^2+^ importation to mitochondria. However, how this protein regulates changes in redox homeostasis in CGL2 subjects needs more investigation.

Since mtDNA lesions were higher and upregulation of *NRF2* mRNA was found in CGL2 subjects, Craveiro-Sarmento *et al.* (2019) investigated whether the BER pathway could be regulated in blood leukocytes. These cells displayed higher mRNA levels of APEX1, OGG1, and OGG1α, and the latter is expressed both in the nucleus and mitochondria and has an essential role in the maintenance of mitochondrial functions (Lia *et al.*, 2018). Table 2 summarizes the main findings of this topic.

### The *CAV1* gene and a severe neonatal progeroid and lipodystrophy syndrome

Whole blood from a subject harboring the heterozygous pathogenic variant c.479_480delTT and c.51_52insGTC in the *CAV1* was associated with a severe neonatal progeroid and lipodystrophy syndrome. The 3-year-old patient also presented a heterozygous variant c.51_52insGTC in the *AGPAT2* gene. The contribution of the latter to the development of this lipodystrophic progeroid disease is unclear. The 3-year-old patient showed severe loss of sWAT, progeroid features, and high levels of TGs in infancy. Fibroblasts isolated from this subject displayed lower levels of the caveolin-1 protein relative to the controls. RNA-seq analysis suggested a downregulation of *LMNA*, *ATM*, *RECQL4*, and *WRN* genes in the whole blood cells from this subject. Furthermore, the Fanconi anemia pathway was also downregulated. However, experimental data were not conducted, and a list with all differentially expressed genes was not provided to confirm these findings. Table 2 summarizes the main findings of this topic.

## Critical roles of DNA damage and repair in adipose tissue homeostasis

The role of DNA repair enzymes in adipose tissue homeostasis was also studied in obesity, revealing the importance of DNA integrity for maintaining the functions of WAT. In this section, we will highlight the main findings concerning the role of NEIL1 (Nei like DNA glycosylase 1) and OGG1 DNA glycosylases, from the BER pathway; ATM, which is involved with the repair of DSBs; and XPV, the DNA polymerase eta that acts bypassing the UV-induced DNA lesions, being involved with damage tolerance by translesion synthesis (Menck and Munford, 2014). Table 3 shows the main findings of this section.

**Table 3 -:** Genes related to DNA repair, DDR, or translesion DNA synthesis and their critical roles in adipogenesis.

Gene code (*OMIM code)	Protein name	Cell model	Changes in redox homeostasis and DNA repair	Adipose tissue and metabolic commitments	Reference
*NEIL1* (*608844)	Endonuclease 8-like 1	*Neil1* ^*-/-*^ mice in the absence of exogenous oxidative stress. *Neil1* ^*-/-*^ mice under chow diet ad libitum.	Increased mitochondrial DNA (mtDNA) damage, especially in male *Neil1* ^*-/-*^ mice.	Severe obesity, dyslipidemia, and hepatic steatosis. Hyperleptinemia and high levels of triglycerides and insulin.	(Vartanian *et al.*, 2006)
*NEIL1* (*608844)	Endonuclease 8-like 1	*Neil1* ^*-/-*^ mice in the absence of exogenous oxidative stress. *Neil1* ^*-/-*^ mice under high-fat diet (HFD).	Increased hepatic expression of inflammatory genes. Reduction in mitochondrial DNA.	Increased body weight and body fat accumulation, hypertriglyceridemia, and glucose intolerance. Hepatic lipid accumulation.	(Sampath *et al.*, 2011)
*OGG1* (*601982)	8-oxoguanine DNA glycosylase	*Ogg1* ^*-/-*^ mice under high-fat diet (HFD).	Downregulation of *Ppargc1a, Ppargc1b* and genes related to TCA cycle and fatty acid oxidation in the liver of *Ogg1* ^*-/-*^ mice.	Higher adiposity, hepatic steatosis, impaired glucose tolerance, and higher levels of insulin and hepatic triglycerides (TGs).	(Sampath *et al.*, 2012)
*OGG1* (*601982)	8-oxoguanine DNA glycosylase	*Ogg1* ^*-/-*^ mice under high-fat diet (HFD).	Increased expression levels of fatty acid oxidation (FAO), lipid uptake, and TCA genes, Higher gene and protein levels of the mitochondrial fission proteins *Drp1* and *Fis1*. No changes in 8-oxoG levels.	Increased lipid deposition in muscle.	(Vartanian *et al.*, 2017)
*OGG1* (*601982)	8-oxoguanine DNA glycosylase	Epididymal adipocytes (eWAT) from transgenic mice targeting OGG1 to mitochondria (*Ogg1* ^*Tg*^ mice).	Bigger mitochondria and higher expression levels of *Pgc1α, Sirt1*, *Tnfα*, *Ikkβ*, and the mitochondrial fusion proteins *Mfn1*, *Mfn2*, and *Opa1*. eWAT from *Ogg1* ^*Tg*^ mice seem to have lower 8-oxodG levels.	OGG1 confers protection against diet-induced obesity, insulin resistance, and adipose tissue inflammation. Decreased body composition and smaller adipocytes in eWAT in *Ogg1* ^*Tg*^ mice under a high-fat diet (HFD). Low levels of glucose, insulin, TGs, and cholesterol in plasma and low levels of TGs and cholesterol in the liver. High expression levels of FAO genes. Lower levels of leptin and higher levels of adiponectin in plasma.	(Komakula *et al.*, 2018)
*OGG1* (*601982)	8-oxoguanine DNA glycosylase	Mouse 3T3-L1 preadipocytes expressing hOGG1a with a mitochondrial targeting sequence (MTS) and preadipocytes from *Ogg1* ^*-/-*^ and *Ogg1* ^*Tg*^ mice.	Increased OGG1 expression and activity during adipogenesis induction, and higher PAR levels in 3T3-L1 mouse preadipocytes expressing MTS-hOGG1a.	Attenuated expression of genes related to preadipocyte differentiation (*Scd1*, *Pparγ*, and *c/ebpα*) and reduced lipid accumulation in preadipocytes from *Ogg1* ^*Tg*^ mice and 3T3-L1 cells expressing -MTS-hOGG1a. Increased expression of genes related to preadipocyte differentiation (*Scd1*, *Pparγ*, and *c/ebpα*) and enhanced lipid accumulation in preadipocytes from *Ogg1* ^*-/-*^ mice.	(Komakula *et al.*, 2021)
*XPV* (*603968)	DNA polymerase eta - POLH	*polη* ^*-/-*^ mice in the absence of exogenous oxidative stress. *polη* ^*-/-*^ mice under a high-fat or fructose diet.	Increased DNA damage, upregulation at both the protein and mRNA levels, and phosphorylation of ATM, H2AX, p21, and p53. Upregulation at both the protein and mRNA levels of NF-κB and PARP-1.	Severe obesity, high levels of insulin, leptin, and HOMA-IR. Increased levels at both the protein and mRNA levels of the adipogenic master regulators PPARγ and SREBP1. Adipocyte senescence.	(Chen *et al.*, 2015)
*ATM* (*607585)	Ataxia telangiectasia serine/threonine kinase	*Atm* ^*+/-*^ mice and *Atm* ^*+/-*^ /ApoE^-/-^) mice under a high-fat diet (HFD).	Delayed activation of Chk-2 and p53. Increased levels of ROS and mtDNA damage in *Atm* ^*+/-*^ mice.	Obesity, hypertension, macrophage infiltration, and hyperlipidemia in *Atm* ^*+/-*^ mice.	(Mercer *et al.*, 2010)
*ATM* (*607585)	Ataxia telangiectasia serine/threonine kinase	*Atm* ^*-/-*^ mice in the absence of exogenous oxidative stress. *Atm* ^*-/-*^ mice under a high-fat diet (HFD).	-	Lipodystrophic-like phenotype (loss of sWAT), insulin resistance, and low levels of serum leptin and adiponectin.	Takagi *et al.*, 2015

OMIM: Online Mendelian Inheritance in Man.

### The role of NEIL1

NEIL1 was one of the first BER enzymes associated with metabolic complications (Vartanian *et al.*, 2006). Under chow diet ad libitum, *Neil1* ^*-/-*^ mice displayed severe obesity, dyslipidemia, and hepatic steatosis. These mice exhibited hepatic steatosis, hyperleptinemia, and high levels of TGs and insulin in plasma. Besides, they found increased mitochondrial DNA (mtDNA) damage and deletions, especially in male Neil1-/- mice (Vartanian **et al.*,* 2006). In another study by the same group, *Neil1* ^*-/-*^ mice under chronic oxidative stress induced by a high-fat diet (HFD) displayed increased body weight and body fat accumulation, HTG, and glucose intolerance (Sampath *et al.*, 2011). They also observed an increased hepatic expression of inflammatory genes and a reduction in mitochondrial DNA. These data demonstrated the role of NEIL1 DNA glycosylase in adipose tissue accumulation and mitochondrial dysfunction.

### The role of OGG1

The role of the OGG1 BER enzyme in metabolic homeostasis has also been investigated by the Lloyd and Sampath groups (Sampath *et al.*, 2012; Vartanian *et al.*, 2017; Komakula *et al.*, 2018, 2021). They first found that *Ogg1* ^*-/-*^ mice were more susceptible to obesity and metabolic dysfunction relative to control mice. Under a high-fat diet (HFD), they presented higher adiposity, developed hepatic steatosis, and showed higher levels of insulin and hepatic TGs. Analysis of microarray and qPCR revealed that genes related to the TCA cycle and FAO were downregulated in the liver of *Ogg1* ^*-/-*^ mice, as well as the *Ppargc1a* and *Ppargc1b* genes that codify to the PPAR-gamma coactivator-1 alpha (*Pgc1α)* and PPAR-gamma coactivator-1 beta (*Pgc1β),* respectively (Sampath **et al.*,* 2012). 

Later, they verified that skeletal muscle from *Ogg1* ^*-/-*^ mice show increased lipid deposition, which included TGs, cholesterol esters (CE), diacylglycerol (DAG), free fatty acids (FFAs), and phospholipids (PLs). Further, gene and protein expression of *Drp1* and *Fis1* proteins, which are associated with mitochondrial fission, were higher in muscle from *Ogg1* ^*-/-*^ mice. Besides, the expression levels of genes regulating FAO and lipid uptake, as well as TCA, were increased relative to WT mice. No differences in 8-oxoG levels were found (Vartanian *et al.*, 2017). 

The contribution of mitochondrial OGG1 to metabolic syndrome was also investigated. Using preadipocytes from transgenic mice targeting OGG1 to mitochondria (*Ogg1* ^*Tg*^ mice), they found a protective role of OGG1 against diet-induced obesity, IR, and adipose tissue inflammation (Komakula *et al.*, 2018). They observed a decreased body weight, fat body composition, and smaller adipocytes in eWAT in *Ogg1* ^*Tg*^ mice under HFD. Furthermore, *Ogg1* ^*Tg*^ mice displayed low levels of glucose, insulin, TGs, and cholesterol in plasma, as well as low levels of TGs and cholesterol in the liver, suggesting that the reduced fat mass observed in *Ogg1* ^*Tg*^ mice does not result in lipodystrophic lipid accumulation in the liver. eWAT of *Ogg1* ^*Tg*^ mice under HFD also exhibited high expression levels of *Pgc1α, Sirt1*, *Tnfα*, *Ikkβ*, and FAO genes, such as *Cpt-1*, *Acox*, *Hsl*, *Atgl*, and *Pparα*. Lower levels of leptin and higher levels of adiponectin were also found in *Ogg1* ^*Tg*^ mice plasma. Since they previously found a downregulation in *Pgc1α* in *Ogg1* ^*-/-*^ mice (Vartanian *et al.*, 2017), the higher levels of this transcriptional co-activator from *Ogg1* ^*Tg*^ mice indicate the role of OGG1 in promoting the mitochondrial metabolism in eWAT. Additionally, since SIRT1 regulates adiponectin levels (Qiang *et al.*, 2007), and both are increased in eWAT of *Ogg1* ^*Tg*^ mice, this work also demonstrated the importance of mtOGG1 for activating the SIRT1-adiponectin axis. They also investigated whether targeting OGG1 to mitochondria changes mitochondrial morphology. They found that mitochondrial are elongated in eWAT of *Ogg1* ^*Tg*^ mice and these mice presented higher expression levels of mitochondrial fusion proteins, such as *Mfn1*, *Mfn2*, and *Opa-1*. Although 8-oxoG levels seem to be reduced in eWAT of *Ogg1* ^*Tg*^ mice under HFD, no statical differences were observed relative to WT mice. Together, these data demonstrate the metabolic protective role of targeting OGG1 to mitochondria in eWAT.

The role of OGG1 in adipogenesis and lipid accumulation was investigated (Komakula *et al.*, 2021). Preadipocytes from *Ogg1* ^*-/-*^ mice displayed increased expression of genes related to preadipocyte differentiation (*Scd1*, *Pparγ*, and *c/ebpα*) and enhanced lipid accumulation. On the contrary, mouse 3T3-L1 preadipocytes from *Ogg1* ^*Tg*^ mice and 3T3-L1 cells expressing-MTS-hOGG1a showed attenuated expression of genes related to preadipocyte differentiation (*Scd1*, *Pparγ*, and *c/ebpα*) and reduced lipid accumulation. Since OGG1 activates PARP-1 (Noren Hooten *et al.*, 2011), and PARylation inhibits adipogenesis (Devalaraja-Narashimha and Padanilam, 2010; Luo *et al.*, 2017), they assessed the role of OGG1 on PARylation in mouse preadipocytes. While PARP-1 protein levels were higher before starting adipocytes differentiation, its levels decreased during adipogenesis induction in both 3T3-L1 cells (expressing-MTS-hOGG1a and GFP-controls), which in accordance with reduced PAR levels. However, MTS-hOGG1a cells exhibited higher PAR levels in all time points of adipocytes differentiation relative to control cells. Increased total protein PARylation was also verified in differentiated primary adipocytes and adipose tissue protein extracts from *Ogg1* ^*Tg*^ mice, whereas primary adipocytes, adipose tissue extracts, liver, and BAT from *Ogg1* ^*-/-*^ mice exhibited reduced levels of total protein PARylation. These findings reveal the role of OGG1 in promoting PARP-1 activity in mice. More data are needed to clarify the contribution of OGG1 in human adipogenesis.

### The role of XPV

The XP-V gene encodes polymerase η (Pol η), which plays a crucial role in preventing UV radiation-induced DNA damage (5). Defects in the gene encoding to pol η produce the variant form (V type) of the autosomal recessive disease Xeroderma Pigmentosum (XP-V) (Masutani *et al.*, 1999). XP-V patients tend to have high sensitivity to UV radiation, which often leads them to develop skin cancer (Masutani **et al.*,* 1999). Chen and co-workers demonstrated that polymerase η deficiency in mice (*polη* ^*-/-*^ mice*)* causes obesity with visceral fat accumulation, hepatic steatosis, hyperleptinemia, hyperinsulinemia, and glucose intolerance. Hypertrophy of adipocytes, high levels of adipogenic regulator genes, such as SREBP1 and PPARγ, infiltration of macrophages, and the presence of CL structures were apparent in *polη* ^*-/-*^ mice.

Comparisons between healthy and pol η-deficient mice showed that *polη* ^*-/-*^ mice had higher levels of DNA damage and greater DDR, due to upregulation and phosphorylation of ATM, H2AX, p21, and p53, as well as upregulation of NF-κB and PARP-1 (Chen *et al.*, 2015). Further, *polη* ^*-/-*^ mice also displayed increased DSBs. It was also found that *polη* ^*-/-*^ mice under a high-fat diet, which induces oxidative stress, showed a DNA-damage mediated senescence. Besides, treatment with a p53 inhibitor, pifithrin-α (PFT-α), reduced adipocyte senescence and attenuated the metabolic abnormalities. (Chen **et al.*,* 2015). On the contrary, DNA damage attenuation induced by N-acetylcysteine (NAC) or metformin antioxidants ameliorated cellular senescence and metabolic abnormalities. These results indicate that high levels of DNA damage are responsible for promoting adipocyte senescence, playing a crucial role in the development of obesity and IR (Chen **et al.*,* 2015). These data revealed the involvement of the DNA lesion bypass polymerase Pol η to protect against metabolic comorbidities.

### The role of ATM

Ataxia-telangiectasia was first described in 1941 by Madam Louis-Bar as a disease characterized by progressive cerebellar ataxia followed by oculocutaneous telangiectasia. In 1957, Boder and Sedgwick reported the disease in seven patients, pointing to a family tendency and frequent pulmonary infection as less marked characteristics of the disease. In the same year, Wells and Shy founded an association between subcutaneous telangiectasia with progressive familial choreoathetosis. The disease caused a significant disorder in the central nervous system, which was initially overshadowed by pulmonary infections (Silberpfennig *et al.*, 1941). Furthermore, ataxia-telangiectasia subjects display DM and IR (Bar **et al.*,* 1978; Blevins and Gebhart, 1996; Morio *et al.*, 2009). 

The ataxia-telangiectasia mutated (*ATM*) gene encodes to the ATM protein, a kinase of 350 kDa that plays a crucial role in DNA repair and is necessary for genomic homeostasis maintenance (Mercer *et al.*, 2010). DSBs activate ATM, which phosphorylates its substrates (or targets) downstream, promoting DNA repair. The main ATM targets are H2AX, cycle cell checkpoints kinases Chk-1 and Chk-2, and the p53 tumoral suppressor gene (Mercer **et al.*,* 2010; Takagi *et al.*, 2015). Although ATM is better characterized as a DDR gene, recent studies point out that defective ATM causes atherosclerosis and metabolic abnormalities. Using an apolipoprotein/ATM heterozygous (*Atm* ^*+/-*^ /ApoE^*-/-*^ ) mice, Mercer and co-workers revealed that *Atm* ^*+/-*^ /ApoE^*-/-*^ mice displayed accelerated atherosclerosis and multiple phenotypes of metabolic syndrome (Mercer **et al.*,* 2010). Further, *Atm* ^*+/-*^ mice were fat, hypertensive, macrophage infiltration, and showed hyperlipidemia under HFD. Fat accumulation and macrophage infiltration were also verified in Atm+/-/ApoE-/- mice. VSMCs from *Atm* ^*+/-*^ mice showed higher DNA fragmentation induced by the prooxidant t-BHP, higher levels of p-ATM and γ-H2AX relative to *Atm* ^*+/+*^ mice, and presented a delayed activation of Chk-2 and p53, but not Chk-1 (Mercer **et al.*,* 2010). Furthermore, increased levels of ROS and mtDNA damage in *Atm* ^*+/-*^ mice were found.

Taken together, Mercer and co-workers observed that ATM haploinsufficiency results in DNA damage in cells that compose atherosclerotic plaques, in addition to accelerating atherosclerosis *in vivo*, and inducing several features of metabolic syndrome and mitochondrial dysfunction (Mercer **et al.*,* 2010). Therefore, defective ATM or its haploinsufficiency causes DNA damage, speeds up atherosclerosis and metabolic syndrome features, and may cause failure in DNA repair and p53 activation, resulting in the reduction of apoptosis and cycle cell interruption (Mercer **et al.*,* 2010).

CCAAT/enhancer binding protein α (C/EBPα) and PPARγ are considered the central regulator for adipocyte differentiation. When PPARγ is activated by an agonist in fibroblasts, a complete differentiation program is stimulated, leading to morphological changes, accumulation of lipids, and the expression of almost all characteristic genes of adipocytes (Rosen and Spiegelman, 2000). Another study revealed that ATM is activated during adipogenesis, besides DNA damage and insulin stimulation, and controls this process via transcriptional regulation of C/EBPα and/or PPARγ, which are required for a complete adipocyte maturation (Takagi *et al.*, 2015). Neither lipid accumulation nor adipocyte differentiation occurred in embryonic fibroblasts of *Atm* ^*-/-*^ knockout mice since there was a defective induction of C/EBPα and PPARγ ATM-dependent expression (Takagi **et al.*,* 2015). Besides, it was observed that *Atm* ^*-/-*^ mice were insulin resistant, presented lower levels of adiponectin and leptin, had less subcutaneous and interscapular adipose tissue, increased visceral fat level (similar to metabolic syndrome), and glucose intolerance when compared to normal *Atm* ^*+/+*^ mice (Takagi **et al.*,* 2015). Finally, it is worth mentioning the importance of adipose tissue for glucose homeostasis, considering that adipokines such as adiponectin, leptin, visfatin, and omentin increase insulin sensitivity, while hypertrophic adipocytes secrete resistin and Tumor Necrosis Factor-alpha (TNFα), which decrease sensitivity to insulin (Rosen and Spiegelman, 2006). Therefore, ATM deficiency leads to impaired adipocyte differentiation, which impairs adipokine secretion, resulting in IR and glucose intolerance (Takagi **et al.*,* 2015). These data revealed the ATM in the regulation of fat metabolism. However, the contribution of DNA damage accumulation and repair in *Atm* ^*-/-*^ mice remains to be determined.

## Interactome analysis of DNA repair- and lipodystrophy-related Genes

**Figure 3 -:**
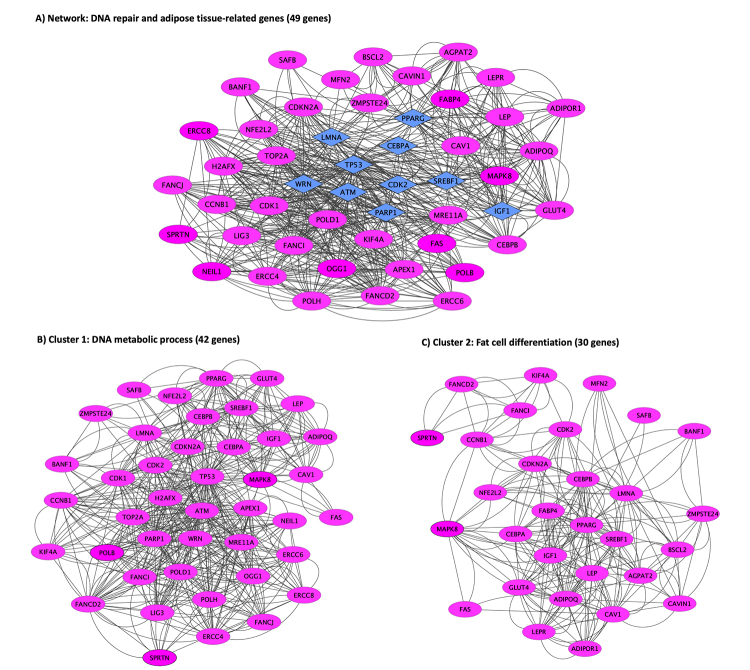
Network comprising the DNA repair- and lipodystrophy- related genes reviewed in this paper. A) PPI network of 49 genes showed high connectivity (676 interactions) between genes involved with DNA repair and adipose tissue. The STRING parameters for the *Homo sapiens* were: Experiments; Database; Neighborhood; and Textmining. The minimum required interaction score was: medium confidence (0.400). Ten hub-bottlenecks (in blue) were depicted by CentiScaPe CytoScape plug-in: *LMNA*, *WRN*, *TP53*, *ATM*, *PARP1*, *PPARG*, *CEBPA*, *CDK2*, *SREBF1*, and *IGF1*. Two clusters of the main network were depicted by MCODE CytoScape plug-in, and their Gene Ontology (GO) was obtained by BiNGO CytoScape plug-in. Cluster 1 (B) was composed of 42 genes and was associated with the DNA metabolic process, while cluster 2 (C) was formed by 30 genes and is related to fat cell differentiation.

**Figure 4 -:**
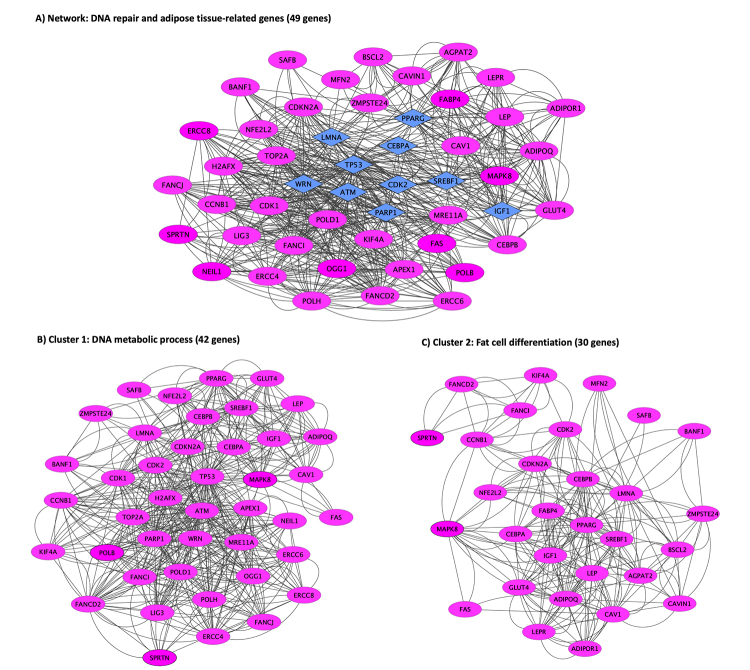
Common and specific genes of cluster 1 (DNA metabolic process) and cluster 2 (fat cell differentiation) and their main transcription factors (TFs). A) InteractiVenn showed that 23 genes from the network are common to cluster 1 (in orange) and cluster 2 (in green), ratifying the crosstalk between proteins first described to have functions associated with DNA repair or adipogenesis. B) The main TFs (in green) regulating genes (in pink) from cluster 1 include: *FOXM1*, *NF-YA*, *SIN3A*, and *E2F4*. C) The main TFs that regulate genes (in pink) from cluster 2 were: *CEBPB*, *POLR2A*, *ATF4*, and *JUN*. The main TFs (in green) that regulate genes of both clusters were found using the iRegulon CytoScape plug-in.

To better clarify the interplay between the altered DNA repair pathways reviewed here and the lipodystrophies’ cell models associated with these DNA repair changes, we performed some systems biology analysis. The interactions of the main proteins described in this review were analyzed using STRING database (Szklarczyk *et al.*, 2017), Cytoscape desktop application (Shannon *et al.*, 2003) and its plugins: Molecular Complex Detection (MCODE) (Bader and Hogue, 2003), CentiScaPe (Scardoni *et al.*, 2009), Biological Networks Gene Ontology (BiNGO) (Maere *et al.*, 2005), and iRegulon (Heberle *et al.*, 2015), and InteractiVenn web tool (Janky *et al.*, 2014). The network containing 49 proteins was firstly built using STRING, which collects and integrates physical (direct) and functional (indirect) interactions. Later, the network was analyzed using Cytoscape. CentiScaPe was used to identify centrality parameters, determining the network nodes that are experimentally and topologically relevant. The protein-protein interactions (PPI) from the network revealed 676 interactions between DNA repair and lipodystrophic proteins (Figure 3A). Two protein clusters (densely connected regions) were detected by MCODE: one cluster had 42 nodes and 580 interactions, and the gene ontology (GO) determined by BiNGO was DNA metabolic process (Figure 3B). The second cluster had 30 genes and 258 interactions, and the BiNGO-determined GO was fat cell differentiation (Figure 3C). CentiScaPe analysis showed that the most dynamic nodes of the network, referred to as hub-bottlenecks (in blue), include: *LMNA*, *WRN*, *TP53*, *ATM*, *PARP1*, *PPARG*, *CEBPA*, *CDK2*, *SREBF1*, and *IGF1*. InteractiVenn analysis revealed that 23 genes from the network are common to Cluster 1 and Cluster 2, ratifying the interplay of proteins from DNA repair and adipogenesis (Figure 4A). It is important to notice that since the STRING network was used as an input to Cytoscape, some experimental data reviewed here were not shown in STRING and, consequently, they were not depicted in the Cytoscape network, such as *PARP1* with *CEBPB*, *BSCL2* with *OGG1*, *APEX1*, and *NFE2L2*. However, even without these data, the network had a significant number of PPI. To scrutinize the regulators of the network, iRegulon was used to find the main transcription factors (TFs) regulating the genes of the network. The TFs controlling cluster 1 (DNA metabolic process) were: *FOXM1*, *NF-YA*, *SIN3A*, and *E2F4* (Figure 4B). The role of *FOXM1* in DNA repair, cell proliferation, and tissue homeostasis was previously described in different works (Tan *et al.*, 2007; Kwok *et al.*, 2010; Millour *et al.*, 2011; Zhang *et al.*, 2012; Monteiro *et al.*, 2013; Khongkow *et al.*, 2014; Zona *et al.*, 2014). NF-YA role in DNA damage/repair was also verified (Jin *et al.*, 2001; Lee *et al.*, 2004; Lin *et al.*, 2014). Besides, *SIN3A* is associated with genomic integrity, and DNA damage (McDonel *et al.*, 2012), and the role of *E2F4* in cell cycle progression was also shown (Ren *et al.*, 2002). Furthermore, the TFs that regulate cluster 2 (fat cell differentiation) were: *CEBPB*, *ATF4*, *JUN*, and *POLR2A* (Figure 4C). The role of these TFs in adipogenesis was previously shown (Yu *et al.*, 2014; Guo *et al.*, 2015; Lee **et al.*,* 2016; Bradford *et al.*, 2019; Ahmed *et al.*, 2019; Ambele *et al.*, 2020; Bléher *et al.*, 2020).

Data reviewed here and the interactomes shown in Figures 3 and 4 reveal a vigorous connection between DNA repair and adipose tissue-related genes. However, how this PPI affects the functions of these genes in the context of adipocyte differentiation has yet to be investigated. Further, the role of the abovementioned TFs in the regulation of this PPI remains to be elucidated. Therefore, lipodystrophies can be a useful model for studying the mechanisms that link genome instability, metabolic dysregulation, and aging.

## Concluding remarks and future directions

Over recent years, advancements in our understanding concerning the genetics of congenital lipodystrophies led to a better knowledge of the onset and progression of these rare diseases. This review highlighted several findings showing the interplay between genes associated with DNA repair and adipogenesis. Based on the many results reviewed here, we concluded that the maintenance of genomic integrity and an effective DNA repair contribute to adipose tissue homeostasis. Therefore, the treatment strategies of congenital lipodystrophies should focus on the elimination/reduction of DNA damage accumulation, as well as on antioxidant therapies.

Furthermore, some questions require more investigation. What is the link between genome stability and metabolism? How does DNA repair deficiency result in several forms of progeroid syndromes with lipodystrophy? How do lipodystrophies caused by pathogenic variants in adipose tissue-related genes result in DNA repair activation? To respond to these questions, it is crucial to scrutinize the DNA repair contributions in different adipose tissue depots obtained from adipose tissue-proficient and lipodystrophic cellular models.
